# Aquatic exercise interventions in the treatment of musculoskeletal upper extremity disorders: A scoping review

**DOI:** 10.1177/02692155251315078

**Published:** 2025-02-02

**Authors:** Lynn Murray, Michelle Kennedy, Michael Malone, Lyn Mair, Lyndsay Alexander

**Affiliations:** 1 Musculoskeletal Physiotherapy Services, 1015 NHS Grampian, Aberdeen, UK; 2 School of Health Sciences, 1018 Robert Gordon University, Aberdeen, UK; 3 Neurological Physiotherapy Services, 1015 NHS Grampian, Aberdeen, UK; 4 Library & Knowledge Services, 1015 NHS Grampian, Aberdeen, UK; 5 The Scottish Centre for Evidence-based, Multi-professional Practice: A JBI Centre of Excellence, Aberdeen, UK

**Keywords:** Aquatic therapy, arm injury, hydrotherapy, physiotherapy, shoulder, rotator cuff, rehabilitation

## Abstract

**Objective:**

To identify literature on aquatic exercise therapy used to manage upper extremity musculoskeletal disorders and identify key concepts, intervention components, and gaps in the evidence base.

**Data sources:**

The comprehensive search included MEDLINE (Ovid), CINAHL (EBSCOHost), Embase (Ovid), CENTRAL (Cochrane Central Register of Controlled Trials) databases and grey literature sources.

**Review methods:**

JBI Scoping review methodology guided this review through protocol development, searching, screening, data extraction and analysis. Study Selection included: Participants – Adults with upper extremity musculoskeletal disorders; Concept – Aquatic based exercise therapy; Context – any setting in any very highly developed nation.

**Results:**

The search identified 5045 sources with 68 studies included in the final synthesis. Findings outlined shoulder problems were the most reported upper extremity condition treated (*n* = 78) especially following rotator cuff repair (*n* = 17), followed by the hand and wrist (*n* = 9), and elbow (*n* = 6). Range of movement (*n* = 36) and resistance exercises (*n* = 17) were the most common interventions reported for aquatic therapy, however compliance with reporting guidance across included studies was poor. Sixteen health domains were identified with range of movement (*n* = 21) and pain (*n* = 20) the most common, and 62 outcome measures were reported related to the identified domains. Qualitative aspects of aquatic interventions were evaluated in two papers.

**Conclusion:**

There is a need for more primary experimental and qualitative studies related to the upper extremity and aquatic therapy. Improved reporting quality of aquatic therapy exercise intervention is required as is the need to establish specific core outcome sets and domains in this area.

## Protocol

Alexander, L., Murray, L., Mair, L., Malone, M., & Kennedy, M. (2022, September 1). The Use of Aquatic Physiotherapy in the Treatment of Musculoskeletal Upper Extremity Disorders: A Scoping Review. Retrieved from osf.io/8nxqg

## Introduction

Musculoskeletal conditions affect approximately 1.71 billion people globally, with high-income countries reporting the highest prevalence (441 million cases) followed by the Western Pacific (427 million) and South-East Asia (369 million) regions.^1^ These conditions contribute significantly to disability, accounting for 17% of years lived with disability worldwide. Notably, 160 million adults aged 15–64 would benefit from rehabilitation, predominantly for musculoskeletal issues.^2^ Common conditions include shoulder pain, affecting 26% of the population, and elbow and hand pain, with prevalence rates of 5.6% and 12.3%, respectively.^3–5^ Injuries in young athletes participating in overhead sports are also prevalent.^6^

Aquatic therapy, which includes various water-based interventions, offers several benefits for individuals with musculoskeletal conditions, such as pain reduction, enhanced strength, increased joint mobility, and improved balance.^7,8^ However, clinical guidance on which specific upper limb conditions benefit from this treatment is lacking.

Aquatic therapy can be conducted in both group and individual settings,^9^ utilising a range of treatment techniques, from active to passive exercises and manual therapies.^10,11–13^ Given the rising healthcare costs^14^ and the necessity for evidence-based practice, it is crucial to evaluate the effectiveness of water-based exercise for upper extremity conditions. Prior to evaluating efficacy, it is important to explore the literature to establish whether a subsequent systematic review and meta-analysis can be conducted.^15^

A preliminary search of databases including MEDLINE and the Cochrane Database revealed no ongoing systematic or scoping reviews focused on aquatic therapy for upper extremity musculoskeletal conditions. While past reviews have explored pain relief in various conditions,^16–18^ they have not specifically addressed upper extremity disorders or water-based interventions comprehensively.

This scoping review aimed to identify existing literature on aquatic exercise therapy for managing upper extremity musculoskeletal disorders and to highlight key concepts, intervention components, and research gaps. The following questions guided the review:

1. Which upper extremity musculoskeletal conditions are managed with aquatic exercise therapy in adults, and what concepts are applied?
2. What is the content of the aquatic therapy interventions, including exercise types, duration, session frequency, and total number of sessions?
3. What health domains and outcome measures are utilised to assess the benefits of aquatic therapy?
4. What has been reported regarding the acceptability, experiences, views, barriers, and facilitators related to aquatic exercise for these conditions?

## Methods and analysis

The scoping review was conducted in accordance with the JBI methodology for scoping reviews which is recognised as the most up to date guidance in scoping review methodology.^19^ The protocol for this review was registered on Open Science Framework (Protocol registered OSF on 1^st^ September 2022)^20^ and the review is reported in accordance with the PRISMA Extension for Scoping Reviews.^21^

Population: This review considered any study that included adults over the age of 18, of any gender, with any upper extremity musculoskeletal disorder who had undertaken aquatic exercise therapy interventions in the management of their condition. The term upper extremity was used and is termed as that part of the body that includes the arm, wrist, and hand.^22^

Any musculoskeletal injury, whether acute or chronic, with either a traumatic or non-traumatic onset was considered. Studies including participants with musculoskeletal shoulder impairment secondary to treatment for breast cancer were included if the musculoskeletal condition was the condition of interest rather than lymphoedema.

This review excluded studies evaluating aquatic exercise interventions for those with fibromyalgia, neurological or rheumatological conditions as these conditions are multisystemic and do not specifically relate to the upper extremity.

Concept: Aquatic therapy is the focus of this review and studies reflecting this and similar terms such as hydrotherapy were included. Balneotherapy studies were only included if there was evidence of aquatic exercise interventions. For this review, the term “aquatic exercise therapy” encompassed various water-based interventions, including hydrotherapy and aquatic exercise.^23^

Context: Any setting such as primary care, secondary care, or community locations in any highly developed nation (defined as the top 66 countries in the Human Development Index)^24^ were included for the findings to be relevant to developed nations.

A three-step search strategy was utilised in this review, with an information specialist supporting the development and conduct of the search strategy. Following an initial search of MEDLINE (Ovid) and CINAHL (EBSCOHost) to identify articles on the topic, text words within the titles and abstracts of relevant articles, and their index terms, were used to develop a full search strategy for MEDLINE (Ovid) which was then adapted for each database and information source (Supplementary File 1). A second full search was then conducted across all included databases and sources, and reference lists of all included sources of evidence were screened for additional sources. Authors of included reports were contacted where possible if additional information was required. Reports published in any language that could be translated using Google Translate^TM^ were included and no date limit was applied to the search.

Databases searched included: MEDLINE (Ovid), CINAHL (EBSCOHost), Embase (Ovid) and CENTRAL (Cochrane Central Register of Controlled Trials). Grey literature sources included: Ethos, Networked Digital Library of Theses and Dissertations, and an advanced search of Google^TM^ using modified search terms to look for grey literature (with results limited to Portable Document Format to locate reports and policies as appropriate). Global aquatic therapy Special Interest Groups, who are members of the International Organisation of the Aquatic Physical Therapists subgroup and whose countries are listed in the top 66 countries in the Human Development Index^24^ were contacted with regards to identifying clinical interest journals and opinions or evidence from clinical experts in the field. Special Interest Groups that could be explored in English were included as there was no translation support for non-English language searching for these forums. Searches were conducted on 11^th^ May 2022 and updated on the 2^nd^ May 2023, the 18^th^ April 2024 and the 21^st^ October 2024.

**Figure 1.**
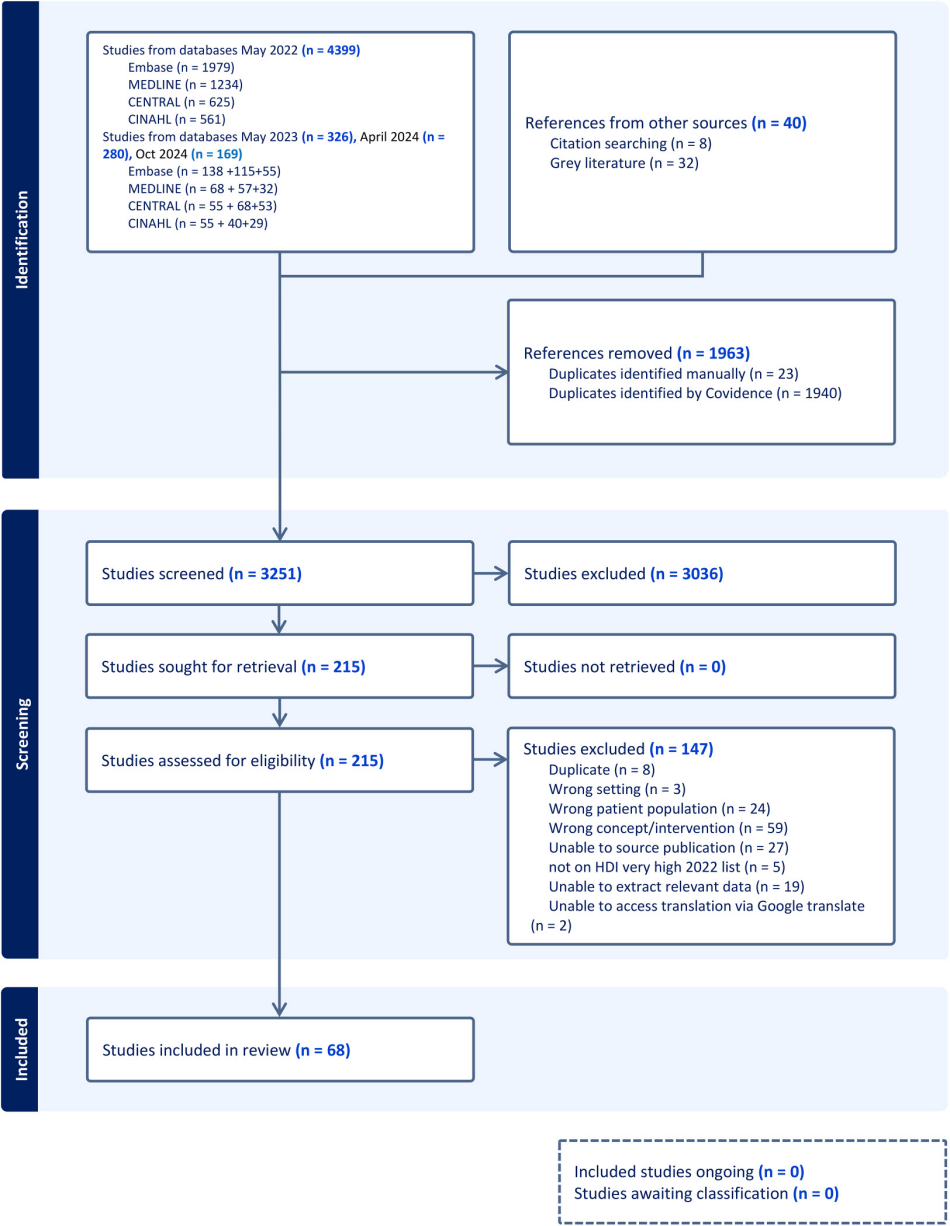
Preferred reporting items for systematic reviews and meta-analyses flowchart (PRISMA).^26^

All identified citations were uploaded to RefWorks (Legacy) to facilitate identification and removal of duplicate citations with Covidence^25^ subsequently used to facilitate screening. Titles and abstracts, then full text sources were independently screened by two reviewers (LA, LMa, MK, MM, LMu) for inclusion in the review. Any sources excluded at full text screening were recorded with reasons for exclusion reported. Title and abstract and full text screening were piloted by the review team prior to starting each stage of the screening process. Any disagreements that arose between the reviewers at each stage were resolved through discussion, or with an additional reviewer. The results of the search and the screening process are presented in a Preferred Reporting Items for Systematic Reviews and Meta-analyses extension for scoping review (PRISMA-ScR) flow diagram (Figure 1).^26^ Systematic reviews were included to identify reviews that had previously been conducted and to allow identification of additional studies that were relevant to this review.

Data was extracted from included papers independently by two reviewers (LMu, LA, MM, MK, LMa) using a customised data extraction tool within Covidence^25^ to facilitate extraction. The tool was piloted on three studies by the review team and amended to ensure that all relevant data could be captured and documented. Extracted data included: the lead author, year of publication, country, population sample size, aim or purpose of the study, study design or source. Participant data included gender, age, condition, ethnicity, comorbidities, time since injury and any other relevant data. Information on the aquatic exercise therapy was extracted and the template for intervention description and replication^27^ was used to capture the intervention content. Health domains and related outcome measures were also extracted as well as results, patient acceptability, experience, views, barriers, and facilitators.

Any disagreements or queries that arose between the reviewers during data extraction were resolved through discussion or by using an additional reviewer. In keeping with scoping review methodology,^19^ no critical appraisal of methodological quality was conducted in this review. Data was exported from Covidence as a Microsoft Excel spreadsheet for subsequent collation and analysis and visual representations developed to aid interpretation of the data.

## Results

The searches identified 5214 records and following screening, 68^28–95^ reports were included in the final scoping review synthesis (see Figure 1). Main reasons for exclusion at full text review included wrong concept/intervention (*n* = 59), incorrect patient population (*n* = 24), unable to extract relevant data (*n* = 19) and duplicate studies (*n* = 8). A reference list of reports excluded at full text review with the reasons for exclusion are presented in Supplementary File 2.

Included reports consisted of 11 systematic reviews,^28–38^ 37 studies (including two trial protocols)^39–74,95^ and 20 text and opinion papers^75–94^ with publication dates ranging from 1986 to 2023 (Supplementary file 3). Reports ranged in type from experimental study designs (*n* = 10, predominantly randomised controlled trials *n* = 8), and observational (*n* = 13, predominantly case reports *n* = 7), to abstracts (*n* = 5), mixed method (*n* = 2), feasibility (*n* = 1), service evaluation (*n* = 1) and a PhD thesis (*n* = 1). Six reviews were not directly related to the role of aquatic exercise therapy on the upper extremity but focused on the broader therapeutic management of musculoskeletal conditions including aquatic therapy. Two reports from one study^67,68^ were identified and considered together in study demographics to avoid duplication of results.

Of the 35 completed studies^39–71,73,74^ identified, 33 were related to people with upper extremity conditions (patients)^39–71^ and two were related to physiotherapists treating the condition.^73,74^ This represented 1480 patient participants, 911 of whom received aquatic exercise therapy. One third (36%) of patient participants did not have gender recorded but where it was stated, there were 242 male and 341 female, with a mean age 56 +/- 9 years, (range 16 to 87 years). Of 347 physiotherapists, one fifth (21.9%) did not have gender reported but where it was stated, there were 67 male and 204 female. One study reported participant ethnicity (Caucasian)^63^ while comorbidities were reported in seven (20%) studies which included other orthopaedic conditions (*n* = 3),^41,49,63^ smoking (*n* = 2),^45,69^ and diabetes (*n* = 2).^43,55^

Studies were conducted in 16 countries predominantly from the USA (*n* = 20)^29,37,41,46,52,55,56,63,75,77,78,81–84,86,88,91,93,94^ and the UK (*n* = 11)^32–34,36,42,58,60,69,70,79,92^ (See Supplementary file 4) although when considered by continent, most studies originated in Europe (48%), North America (37%) or Oceania (6%). Full participant characteristics are presented in Supplementary File 5.

The length of time aquatic exercise interventions lasted (between a one-off session^43^ and weekly/monthly participation for up to 48 semesters (1 semester = 15 weeks^49^) and the time at which they commenced, varied between studies. Aquatic therapy was used in the acute stages following fracture (*n* = 5)^51,53,64,67,68^ and surgical rotator cuff repair (*n* = 7),^39,45,47,48,57,59,71,95^ in the subacute phase after shoulder arthroplasty (*n* = 1)^52^ and in the chronic stages of injury (*n* = 10).^41,42,49,50,55,58,62,63,65,66^ The most common chronic conditions reported included breast cancer related shoulder pain and stiffness (*n* = 3),^42,49,58^ rotator cuff tear (*n* = 2)^41,63^ and frozen shoulder (*n* = 2).^55,65^ One study's participants commenced aquatic exercise therapy less than five months after shoulder fracture^51^ and one study utilised one session of aquatic exercise therapy within 48 h of a manipulation under anaesthetic for frozen shoulder.^43^

Shoulder problems (*n* = 79)^28–36,38–49,51,52,55,57–66,70,71,73,74–78,80,82–95^ were the most frequently treated upper extremity condition, followed by wrist or hand (*n* = 9),^50,53,54,56,67,68,72,79,82^ elbow (*n* = 6)^75,82,83^ and arm conditions (*n* = 1)^69^ reported across all 67 studies (Table 1). The most common shoulder condition was rotator cuff repair (*n* = 17),^33,39,40,45,47,48,57,59,71,77,78,80,84,87,89,91,93,95^ followed by shoulder pain (shoulder pain, injury, pathology, rotator cuff injury, myofascial pain, and breast cancer related shoulder pain) (*n* = 15),^29,31,35,36,38,42,49,58,61,62,70,75,83,84,86^ shoulder fracture (*n* = 8),^28,34,51,60,64,80,82^ rotator cuff tear (*n* = 7),^30,32,41,63,82,90,92^ and shoulder arthroplasty (*n* = 7).^72,76,80,85,87,88,94^ Elbow conditions such as soft tissue injury (*n* = 2),^82^ fracture (*n* = 2)^82,83^ and lateral epicondylitis (*n* = 1)^75^ were noted solely in the text and opinion literature. Various traumatic and chronic wrist and hand conditions were reported including distal radius fractures (*n* = 2),^67,68,72^ complex regional pain syndrome (*n* = 2),^53,79^ and carpal tunnel syndrome (including decompression surgery) (*n* = 2).^56^

**Table 1.** Upper extremity conditions treated using aquatic therapy (all studies).

Condition	Primary paper	Systematic review	Text and opinion	Collective total
*Shoulder conditions*				
Rotator cuff repair	9	1	8	18
Shoulder pain	6	5	4	15
Shoulder fracture	4	2	2	8
Rotator cuff tear	2	2	3	7
Shoulder arthroplasty	1	0	6	7
Frozen shoulder	4	0	1	5
Shoulder stabilisation surgery	1	0	4	5
Rotator cuff related pain	3	0	2	5
Shoulder dislocation/ subluxation/ instability	1	0	3	4
Subacromial decompression	0	0	3	3
Shoulder surgery	0	0	2	2
*Elbow conditions*				
Elbow soft tissue injury	0	0	2	2
Elbow fracture / dislocation	0	0	2	2
Lateral epicondylitis	0	0	1	1
*Wrist and hand conditions*				
Carpal tunnel	2	0	0	2
Chronic Regional Pain Syndrome (CRPS)	1	0	1	2
Wrist and hand injury	1	0	1	2
Hand/finger osteoarthritis	1	0	0	1
Distal radius fracture	2	0	0	2
*Arm conditions*				
Surgical debridement and release axilla, elbow, and arm	1	0	0	1
Nerve palsy	0	0	1	1
Upper extremity orthopaedic injury	0	1	0	1
Pain	0	0	1	1
Post surgery	0	0	1	1
*Total per study type*	38	11	48	

**Figure 2.**
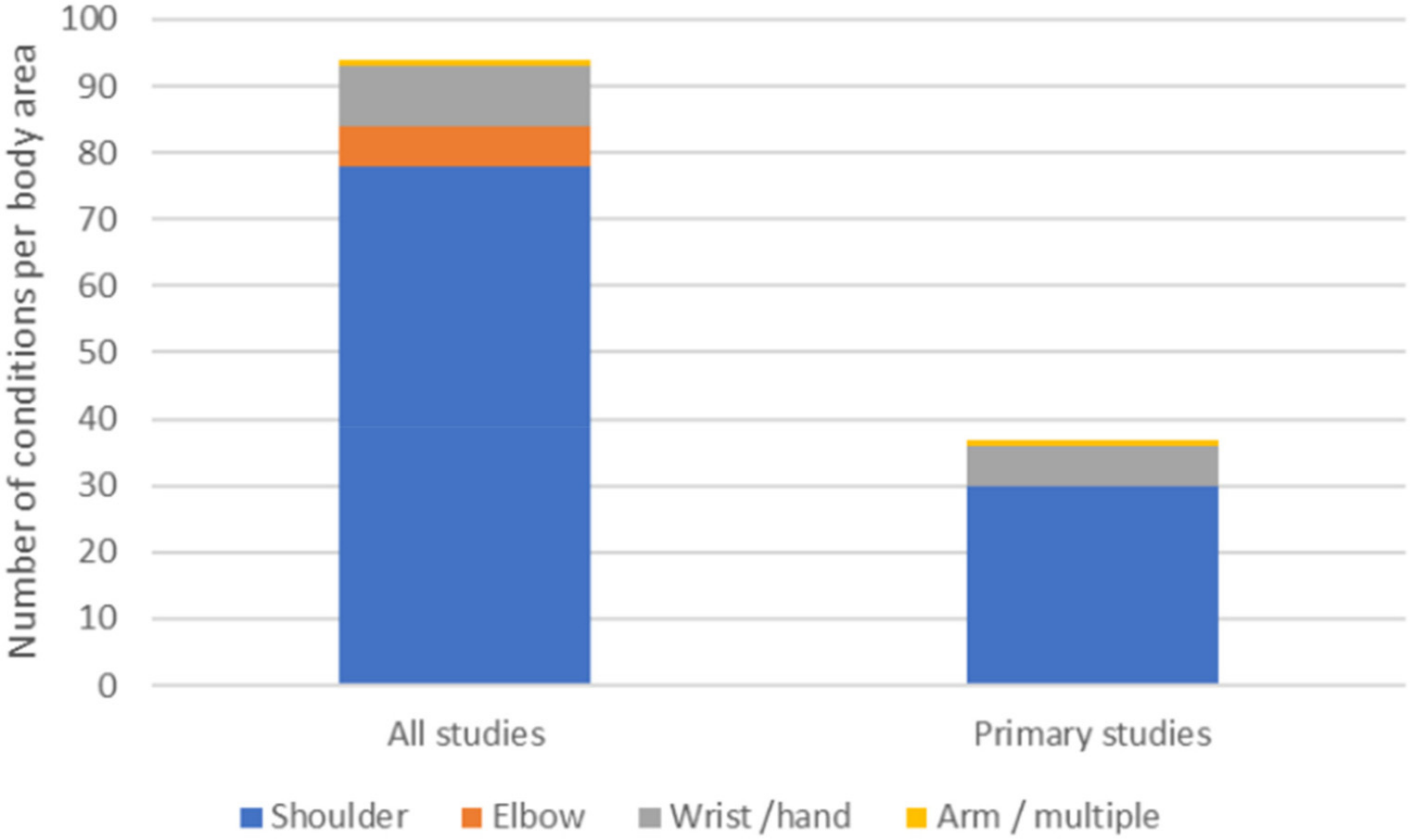
Upper extremity area for all included reports compared to studies alone.

In the 35 studies, 38 upper extremity conditions were treated using aquatic exercise therapy and these were then grouped into 15 different conditions across the studies. Rotator cuff repair was the most evaluated (*n* = 8),^39,40,45,47,48,57,59,71^ followed by shoulder pain (including breast cancer treatment related shoulder pain) (*n* = 6),^42,49,58,61,62,70^ shoulder fracture (*n* = 4),^51,60,64^ frozen shoulder (*n* = 4)^43,44,55,65^ and rotator cuff related shoulder pain (*n* = 3).^66,73,74^ No studies focussed on elbow conditions and six considered wrist and hand conditions.^50,53,54,56,67,68,72^ Overall, the shoulder had the highest number of studies related to aquatic exercise therapy compared to the elbow, wrist, and hand (see Figure 2).

Very few studies explicitly stated they adopted an aquatic exercise therapy concept to guide the intervention. One included deep water running^61^ in the exercise programme and one study included the Bad Regaz Ring Method^10^ in a wider programme of exercise.

Aquatic exercise therapy has multiple terms that are used interchangeably to represent the concept and this review has identified 25 different descriptors (See Supplementary file 6) where hydrotherapy is the most frequently used term (*n* = 15),^34,43,44,51,58,60,74,79,84,85,87,88,90–92^ followed by aquatic therapy (*n* = 13)^29,30,36,40,45,46,48,52,55,70,78,93–95^ and whirlpool (*n* = 4)^53,54,67,68,.72^ Of the primary studies related to patient and physiotherapy participants, the rationale for the use of aquatic therapy was not clearly stated in ten studies.^43,44,55,58,60,64,65,71,73,74^

**Figure 3.**
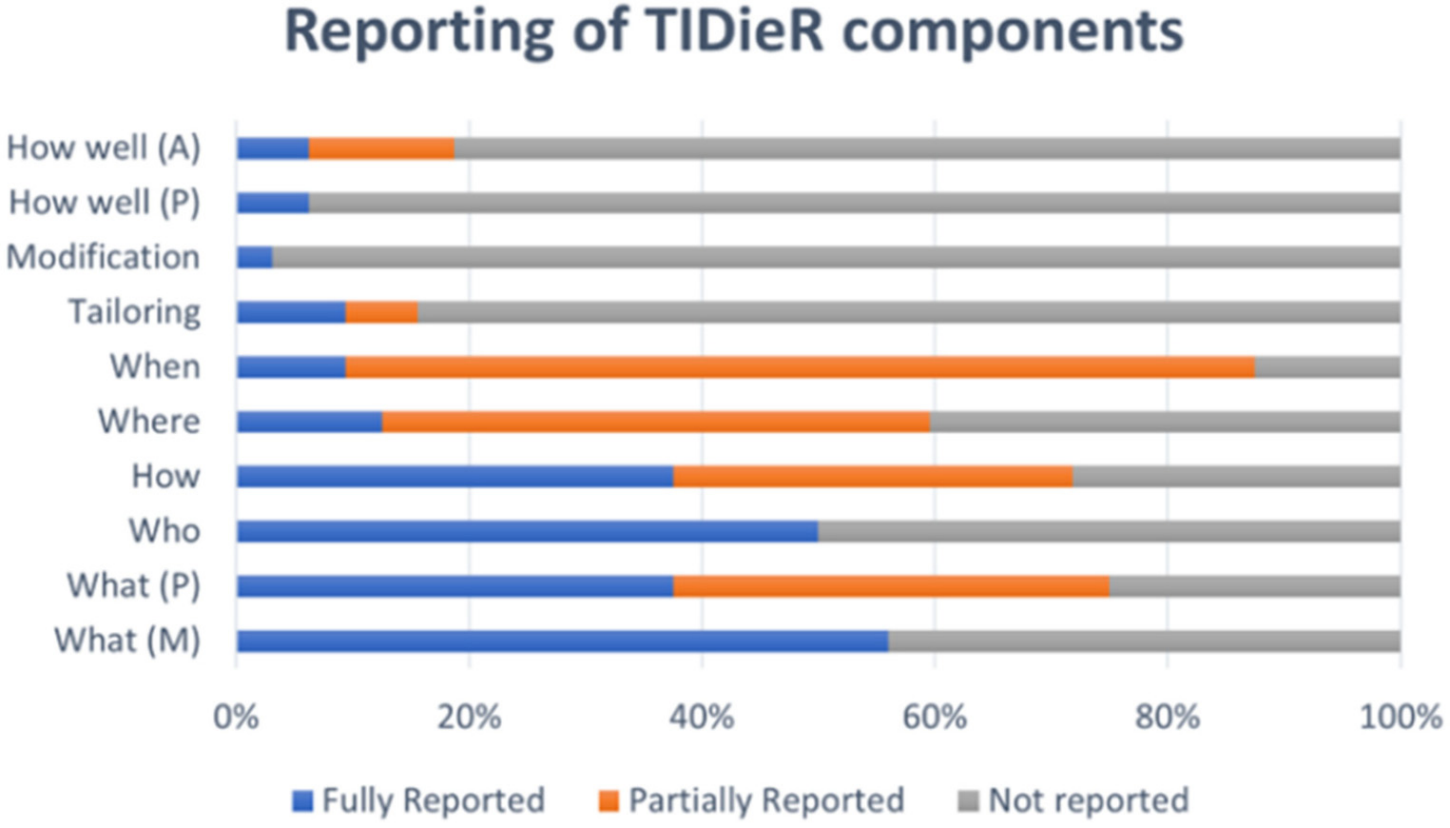
Aquatic exercise therapy intervention reporting compliance.

Data was measured against the template for intervention description and replication^27^ when extracting information regarding aquatic exercise therapy interventions and the compliance with this is recorded in Supplementary file 7 and Figure 3. The studies were graded as fully reported, partially reported (some detail but unable to replicate the component fully) and not reported. Apart from reporting who delivered the intervention (50% of studies) and what materials were used (56% of studies), compliance with the template was poor (less than 50% compliance).

Aquatic exercise therapy interventions were delivered by physiotherapists (*n* = 14)^40–42,45,46,48,49,57,62,63,65–69^ aquatic or fitness instructors (*n* = 2),^49,50^ kinesiologists (*n*-=1)^39^ or therapy assistants (*n* = 1),^41^ with 16 studies not reporting this information.^43,44,47,51–56,58–61,64,70,71^ Two studies noted dual physiotherapist and aquatic therapist roles.^63,66^ Settings were poorly reported with most studies not stating whether sessions were delivered in healthcare or community settings (*n* = 20).^40,42–46,48–50,54–61,64,66,71^ Studies that mentioned a location, had healthcare settings as the most prevalent (*n* = 10),^39,47,51–53,63,65,67,68,69,70^ followed by a combination of community and health care settings (*n* = 2)^41,63^ where participants carried out home aquatic exercise programmes.

Most interventions were delivered face to face (*n* = 24)^39–42,45,46,48–50,52–55,57,58,60–69^ although 13 studies did not report whether this was individually or as part of a group.^41,45,46,48,53,55,57,58,60,63,65,66,69^ Group sessions (*n* = 7),^39,40,42,49,50,61,64^ individual sessions (*n* = 4)^52,54,62,67,68^ and self-directed aquatic programmes in conjunction with face-to-face sessions (*n* = 2)^41,63^ were identified as forms of delivery. Water temperature was recorded in 13 studies with whirlpool temperatures being either 34 °C^62^ or 40 °C^67,68^ and pool temperatures ranging between 27–36.5 °C (*n* = 11).^39,42,45,48–51,57,62,64,66^

A range of equipment (*n* = 32) used to assist intervention delivery for the upper extremity was identified across 18 studies^40–42,45,46,50–54,56,60,62–64,66,67,68,69^ including handheld equipment such as dumbbells (*n* = 4),^41,51,52,66^ aquatic gloves (*n* = 3),^41,51,63^ and noodles (*n* = 3).^41,42,51^ Most aquatic exercise therapy programmes consisted of multiple exercise types and 31 studies described the aquatic intervention. The types of exercises used were categorised as ROM exercises of one or more joint and /or one or more type (active, active assisted, passive) (*n* = 36)^39–42,45,46,48–50,52–54,56,57,59,60,62–64,66,67,68,69^ resistance exercises (*n* = 17)^40–42,45,46,48,49,51,52,57,59,63,64,66,69^ proprioceptive (*n* = 3),^46,51,60^ aerobic (*n* = 3)^49,61,69^ proprioceptive neuromuscular facilitation (*n* = 2),^41,69^ and functional exercise (*n* = 2).^41,52^ A range of adjunct aquatic treatments for shoulder conditions were mapped including manual therapy techniques (*n* = 3),^46,60,62^ relaxation and breathing interventions (*n* = 3),^42,49,51^ and exercises for the trunk and lower extremity (*n* = 3)^44,49,52^ (see Supplementary file 8).

A minority of studies detailed repetitions or sets for exercise interventions (*n* = 7).^39,40,42,53,54,56,63^ One study provided details of sets, repetitions, hold and rest times in relation to aquatic exercise.^53^ Tailoring (*n* = 5)^41,42,51,63,65^ and modification (*n* = 1)^60^ of aquatic exercise programmes were poorly reported in studies with few (*n* = 6)^41,42,51,60,63,65^ reporting any adaptation based on individual need. Adherence and fidelity data were also partially reported in six papers^39–41,45,49,52^ and fully reported in one.^42^

Recording of intervention session frequency varied between studies. Some recorded this as attendances per week or day (*n* = 14),^39,40,45,47,50,51,55,57,60,62,65,67,68,70,71^ others by total number of sessions attended (*n* = 9),^41,43,44,46,54,56,58,63,69^ by attendances per week as well as total sessions (*n* = 7),^42,49,53,59,61,64,66^ or twice weekly sessions (*n* = 8).^39,40,50,53,55,61,64^ The number of sessions recorded for interventions varied between one (*n* = 1)^43^ to 34 (*n* = 1)^59^ provided over a timescale of a one-off session^43^ to seven months.^4^ Length of sessions varied from 11^56^ to 60,^42,59,62^ minutes, with 15 studies not reporting this information.^40,41,43–48,52,57,58,63,66,69,71^ In most studies, aquatic exercise therapy was undertaken as part of a wider programme of care with many participants undertaking land-based exercise programmes (*n* = 21),^39,40,43,45–48,51–53,55,57–60,64–66,69,71^ manual therapy (*n* = 5),^41,53,59,60,65^ electrotherapy (*n* = 2),^65,66^ and other unspecified interventions (*n* = 2).^63,67^ Steroid injection (*n* = 1),^55^ muscle relaxant injection (*n* = 1),^52^ heat pack (*n* = 1),^66^ relaxation (*n* = 1)^51^ and smoking cessation advice (*n* = 1)^69^ were also used as part of wider care programmes.

There were 16 health domains reported across the studies and 62 outcome measures used to evaluate interventions (Supplementary file 9). Range of Movement (*n* = 21),^39–41,43,47,48,52,54,55,57,58–64,66,68,69,71^ and pain (*n* = 20)^41,42,44,47,48,50,52–55,57,59,60,62,64–67,71^ were the most frequently noted domains, followed by function (*n* = 9),^41,48,49,53,57,59,63,64,66^ strength (*n* = 9),^41,50,53,57,61,63,65^ and adverse events or complications (*n* = 7).^41,43,45,47,48,58,59^ It is noted that there were 13 instances where authors had documented outcome measures without clear reference to the domain against which they were measured.^42–46,48,52,58,59,67,69–71^

Visual Analogue Scales were the main outcome measure used to evaluate pain for all upper extremity conditions (*n* = 14)^42,44,48,53,54,57,59,60,62,64–66,71,77^ with the Numerical Pain Rating Scale (*n* = 2),^41,47^ the Shoulder Pain and Disability Index (*n* = 2),^55,66^ Arthritis Impact Measurement Scales 2Short Form (*n* = 1),^50^ and Pain Disability Questionnaire (*n* = 1)^41^ less frequently utilised.

Shoulder joint range of movement was measured passively, actively, and functionally with authors varying the types of movement assessed across studies. Nine studies (27%)^43,47,48,52,55,58,60,61,71^ did not report the outcome measure related to shoulder range of movement. Elbow movement was reported in one paper, but no outcome measure was aligned to this measurement.^69^ Two studies reported the use of goniometry to measure hand or wrist movement using either wrist active range of movement^68^ or total active movement of the finger joints.^54^

Shoulder strength was measured isometrically (*n* = 4)^41,57,63,65^ or isokinetically (*n* = 1)^57^ with hand grip strength reported in four studies principally using handheld dynamometry.^50,53,61,63^ Grip strength was used as an outcome measure for studies related to shoulder conditions (*n* = 2)^61,63^ as well as those related to wrist and hand injuries (*n* = 2).^50,53^

Function (*n* = 9)^41,48,49,53,57,59,63,64,66^ was evaluated using 16 different outcome measures for shoulder function^41,48,49,57,59,63,64,66^ and two outcome measures used in relation to wrist and hand pathology.^53^

Patient acceptability and experience of aquatic exercise therapy for the upper extremity was considered in two mixed methods papers.^49,62^ One paper used in-depth interviews to gather participant views of aquatic exercise therapy for people with chronic musculoskeletal conditions including shoulder pain, and outlined positive opinions related to the broad themes of pain reduction, increased relaxation and relief of tiredness, ease of use, postural awareness and novelty and enjoyment.^62^ The second study explored factors influencing long term participation in aquatic exercise following breast surgery.^49^ Female participants felt arm movements in the pool were easier and less painful compared to daily life and more comfortable compared to other land-based forms of exercise. Water was felt to induce a comfortable feeling, both by relieving the weight and by inducing a light pressure to the affected arm as well as creating positive effects of exercising in warm water related to mobility.^49^

Barriers and facilitators to aquatic exercise therapy for people with upper extremity conditions were not considered by any studies included in this review. One study reported facilitators such as weightlessness leading to reduced pain on exercise and the benefits of social interaction from the group setting as reasons for continued participation in an aquatic post-breast cancer surgery intervention.^49^

## Discussion

This scoping review is the first comprehensive examination of the literature on aquatic exercise therapy for managing upper extremity conditions. Currently, aquatic exercise therapy is primarily integrated into broader therapeutic interventions, particularly focusing on shoulder rehabilitation following rotator cuff repair. Although many studies advocate for aquatic exercise therapy across various conditions,^75–95^ there is a notable lack of homogeneous randomised controlled trials suitable for meta-analysis, especially regarding elbow, wrist, and hand conditions.

Improving the quality of research through standardised reporting guidelines is essential for translating findings into clinical practice.^96–98^ The studies included in this review often demonstrated poor adherence to the template for intervention description and replication (TIDieR) guidelines,^27^ compounded by some studies published pre-template or not peer reviewed. This lack of rigorous reporting mirrors other research,^99^ limits the replicability of interventions and highlights the complexity of exercise interventions.^100^ Future aquatic exercise therapy research should prioritise robust reporting to facilitate clearer comparisons and assessments.

A scarcity of qualitative studies on aquatic exercise therapy for upper extremity conditions has been identified, echoing trends in other physiotherapy research fields.^101^ Qualitative research can provide valuable insights into patient experiences, informing the design of effective interventions.^102,103^ Increased qualitative studies would enhance understanding of health behaviours and patient perspectives regarding aquatic exercise therapy.

This review has also shown that aquatic therapy is often conducted in cooler pool temperatures than the recommended 32–35 °C,^14^ with some studies using temperatures as low as 28°C. While physiological effects have been studied,^104,105^ the impact of water temperature on clinical outcomes for musculoskeletal conditions remains unclear. Additionally, exploring aquatic exercise therapy delivery in community settings, which typically feature cooler pools, could provide insights into long-term self-management of chronic conditions and healthcare cost implications. Understanding the acceptability of cooler water and the differences between community and clinical settings is critical for future implementation.

The relationship between resources utilised and outcomes achieved in healthcare is important to consider.^106^ Although most interventions occurred in healthcare settings, there is a lack of studies assessing cost-effectiveness compared to land-based therapies. Previous analyses suggest significant returns on investment for aquatic therapy services, warranting further evaluation to substantiate these findings.^107,108^

Multiple health domains and outcome measures were employed to evaluate treatment interventions related to upper extremity musculoskeletal conditions and is likely reflective of the integration of aquatic exercise therapy into broader treatment protocols. However, this review did not identify a core outcome set^109^ specifically for upper extremity aquatic interventions, highlighting the need for standardisation to enhance research consistency. The lack of consistency in reported outcome measures complicates the evaluation of treatment efficacy.

Interestingly, key health domains such as fear avoidance and kinesiophobia were not addressed in this review. These factors may be crucial in assessing the value of aquatic exercise therapy, particularly as anecdotal evidence suggests they could influence treatment outcomes. Previous studies have shown aquatic therapy's potential in managing kinesiophobia related to chronic conditions, emphasising the need for further exploration in upper extremity disorders.^110,111^

While this scoping review is robust, some limitations exist, including the potential for missed literature despite a comprehensive search strategy. Access to certain publications was restricted (due to access and translation issues), which may have affected the breadth of the findings.

In conclusion, literature on aquatic exercise therapy for upper extremity disorders primarily focuses on shoulder conditions, particularly in the context of rotator cuff rehabilitation. Aquatic interventions are often delivered as part of larger physiotherapy packages of care, necessitating a need for clearer understanding of the specific benefits offered from the addition of aquatic exercise therapy. Overall, there is a need for more robust experimental and qualitative studies to evaluate the effectiveness of aquatic exercise therapy, optimal timing in recovery, and patient experiences. Additionally, future research should consider health domains such as fear avoidance and kinesiophobia to develop comprehensive clinical recommendations and enhance practice.

Clinical message

• Aquatic exercise therapy is commonly part of a broader care plan, often targeting shoulder issues, especially in early rehabilitation after rotator cuff repair.
• Aquatic interventions frequently include range of motion and resistance exercises for the upper extremities.
• Water temperatures ranged from 27 to 36.5 °C, lower than the UK guidance of 32–35 °C, suggesting community pools may be suitable, but further research is required.
• Both community and healthcare settings provide aquatic therapy, but it's essential to connect clinical outcomes and cost-effectiveness with the setting, water temperature, and session details.

## Supplemental Material

sj-docx-1-cre-10.1177_02692155251315078 - Supplemental material for Aquatic exercise interventions in the treatment of musculoskeletal upper extremity disorders: A scoping review Supplemental material, sj-docx-1-cre-10.1177_02692155251315078 for Aquatic exercise interventions in the treatment of musculoskeletal upper extremity disorders: A scoping review by Lynn Murray, Michelle Kennedy, Michael Malone, Lyn Mair and Lyndsay Alexander in Clinical Rehabilitation

sj-doc-2-cre-10.1177_02692155251315078 - Supplemental material for Aquatic exercise interventions in the treatment of musculoskeletal upper extremity disorders: A scoping review Supplemental material, sj-doc-2-cre-10.1177_02692155251315078 for Aquatic exercise interventions in the treatment of musculoskeletal upper extremity disorders: A scoping review by Lynn Murray, Michelle Kennedy, Michael Malone, Lyn Mair and Lyndsay Alexander in Clinical Rehabilitation

sj-pdf-3-cre-10.1177_02692155251315078 - Supplemental material for Aquatic exercise interventions in the treatment of musculoskeletal upper extremity disorders: A scoping review Supplemental material, sj-pdf-3-cre-10.1177_02692155251315078 for Aquatic exercise interventions in the treatment of musculoskeletal upper extremity disorders: A scoping review by Lynn Murray, Michelle Kennedy, Michael Malone, Lyn Mair and Lyndsay Alexander in Clinical Rehabilitation

sj-pdf-4-cre-10.1177_02692155251315078 - Supplemental material for Aquatic exercise interventions in the treatment of musculoskeletal upper extremity disorders: A scoping review Supplemental material, sj-pdf-4-cre-10.1177_02692155251315078 for Aquatic exercise interventions in the treatment of musculoskeletal upper extremity disorders: A scoping review by Lynn Murray, Michelle Kennedy, Michael Malone, Lyn Mair and Lyndsay Alexander in Clinical Rehabilitation

sj-docx-5-cre-10.1177_02692155251315078 - Supplemental material for Aquatic exercise interventions in the treatment of musculoskeletal upper extremity disorders: A scoping review Supplemental material, sj-docx-5-cre-10.1177_02692155251315078 for Aquatic exercise interventions in the treatment of musculoskeletal upper extremity disorders: A scoping review by Lynn Murray, Michelle Kennedy, Michael Malone, Lyn Mair and Lyndsay Alexander in Clinical Rehabilitation

sj-pdf-6-cre-10.1177_02692155251315078 - Supplemental material for Aquatic exercise interventions in the treatment of musculoskeletal upper extremity disorders: A scoping review Supplemental material, sj-pdf-6-cre-10.1177_02692155251315078 for Aquatic exercise interventions in the treatment of musculoskeletal upper extremity disorders: A scoping review by Lynn Murray, Michelle Kennedy, Michael Malone, Lyn Mair and Lyndsay Alexander in Clinical Rehabilitation

sj-docx-7-cre-10.1177_02692155251315078 - Supplemental material for Aquatic exercise interventions in the treatment of musculoskeletal upper extremity disorders: A scoping review Supplemental material, sj-docx-7-cre-10.1177_02692155251315078 for Aquatic exercise interventions in the treatment of musculoskeletal upper extremity disorders: A scoping review by Lynn Murray, Michelle Kennedy, Michael Malone, Lyn Mair and Lyndsay Alexander in Clinical Rehabilitation

sj-docx-8-cre-10.1177_02692155251315078 - Supplemental material for Aquatic exercise interventions in the treatment of musculoskeletal upper extremity disorders: A scoping review Supplemental material, sj-docx-8-cre-10.1177_02692155251315078 for Aquatic exercise interventions in the treatment of musculoskeletal upper extremity disorders: A scoping review by Lynn Murray, Michelle Kennedy, Michael Malone, Lyn Mair and Lyndsay Alexander in Clinical Rehabilitation

sj-docx-9-cre-10.1177_02692155251315078 - Supplemental material for Aquatic exercise interventions in the treatment of musculoskeletal upper extremity disorders: A scoping review Supplemental material, sj-docx-9-cre-10.1177_02692155251315078 for Aquatic exercise interventions in the treatment of musculoskeletal upper extremity disorders: A scoping review by Lynn Murray, Michelle Kennedy, Michael Malone, Lyn Mair and Lyndsay Alexander in Clinical Rehabilitation
