# Supplementary material for: Aquatic exercise interventions in the treatment of musculoskeletal upper extremity disorders: A scoping review
Source: Clin Rehabil. 2025 Feb 2;39(5):565–79. doi: 10.1177/02692155251315078 (PMC12099020; doi:10.1177/02692155251315078)
Supplement: sj-docx-1-cre-10.1177_02692155251315078 - Supplemental material for Aquatic exercise interventions in the treatment of musculoskeletal upper extremity disorders: A scoping review [file sj-docx-1-cre-10.1177_02692155251315078.docx]

**Aquatic therapy searches run on 11 May 2022 (Medline, Embase, CINAHL, CENTRAL)**

**MEDLINE**

**Database:**
Ovid MEDLINE(R) ALL <1946 to May 10, 2022>

| **#** | **Query** | **Results from 11 May 2022** |
| --- | --- | --- |
| 1 | Hydrotherapy/ | 2,622 |
| 2 | Aquatic Therapy/ | 13 |
| 3 | Balneology/ | 5,898 |
| 4 | (hydrotherap* or balneotherapy or balneology or balneopathy).ab,kf,ti. | 4,250 |
| 5 | ((aqua* adj4 exercis*) or (aqua* adj4 therap*) or (aqua* adj4 physiotherap*) or (aqua* adj4 physical therap*) or (aqua* adj4 rehabilitat*) or (aqua* adj4 train*)).ab,kf,ti. | 1,229 |
| 6 | ((water* adj4 exercis*) or (water* adj4 therap*) or (water* adj4 physiotherap*) or (water* adj4 physical therap*) or (water* adj4 rehabilitat*) or (water* adj4 train*)).ab,kf,ti. | 5,124 |
| 7 | ((aqua* adj4 ai chi) or (aqua* adj4 aichi) or (aqua* adj4 tai chi) or (aqua* adj4 taichi) or (aqua* adj4 running) or (aqua* adj4 aerobic*) or (aqua* adj4 fitness)).ab,kf,ti. | 284 |
| 8 | ((water* adj4 ai chi) or (water* adj4 aichi) or (water* adj4 tai chi) or (water* adj4 taichi) or (water* adj4 shiatsu) or Halliwick* or Burdenko* or "Bad Ragaz*" or watershiatsu or watsu or deep water running or (water* adj4 aerobic*) or (water* adj4 fitness)).ab,kf,ti. | 1,204 |
| 9 | (pool therap* or whirlpool or water bath*).ab,kf,ti. | 5,647 |
| 10 | or/1-9 | 21,042 |
| 11 | upper extremity/ or arm/ or axilla/ or elbow/ or forearm/ or hand/ or fingers/ or thumb/ or metacarpus/ or shoulder/ or wrist/ | 180,479 |
| 12 | "bones of upper extremity"/ or arm bones/ or humerus/ or humeral head/ or radius/ or ulna/ or olecranon process/ or clavicle/ or hand bones/ or carpal bones/ or capitate bone/ or hamate bone/ or lunate bone/ or pisiform bone/ or scaphoid bone/ or trapezium bone/ or trapezoid bone/ or triquetrum bone/ or finger phalanges/ or metacarpal bones/ or scapula/ or acromion/ or coracoid process/ or glenoid cavity/ | 50,095 |
| 13 | shoulder injuries/ or rotator cuff injuries/ or shoulder dislocation/ or shoulder fractures/ or bankart lesions/ or shoulder impingement syndrome/ | 20,966 |
| 14 | arm injuries/ or forearm injuries/ or radius fractures/ or ulna fractures/ or humeral fractures/ or tennis elbow/ or monteggia's fracture/ or colles' fracture/ | 28,933 |
| 15 | shoulder pain/ | 5,531 |
| 16 | deltoid muscle/ or pectoralis muscles/ or rotator cuff/ | 12,294 |
| 17 | acromioclavicular joint/ or elbow joint/ or hand joints/ or carpal joints/ or carpometacarpal joints/ or finger joint/ or metacarpophalangeal joint/ or palmar plate/ or wrist joint/ or triangular fibrocartilage/ or shoulder joint/ | 55,538 |
| 18 | bursitis/ | 3,724 |
| 19 | brachial plexus/ or median nerve/ or musculocutaneous nerve/ or radial nerve/ or ulnar nerve/ | 25,720 |
| 20 | elbow tendinopathy/ | 57 |
| 21 | median neuropathy/ or carpal tunnel syndrome/ or radial neuropathy/ or ulnar neuropathies/ or cubital tunnel syndrome/ or ulnar nerve compression syndromes/ | 11,889 |
| 22 | Wrist Injuries/ | 6,654 |
| 23 | hand injuries/ or finger injuries/ | 18,955 |
| 24 | de quervain disease/ or trigger finger disorder/ | 849 |
| 25 | Dupuytren Contracture/ | 2,847 |
| 26 | (upper extremit* or upper limb* or shoulder* or arm or arms or axilla or forearm* or wrist* or hand or hands or finger* or forefinger* or thumb* or digit or digits).ab,kf,ti. | 969,930 |
| 27 | (humerus or humeral or radius or radial head or ulna or ulnar or ulnal or olecranon or clavicle or hamate or lunate or capitate or pisiform or scaphoid or trapezium or trapezoid or triquetrum or phalan* or metacarp* or carpal or carpus or scapula or acromion or coracoid or glenoid or glenohumeral or radioulnar or radiocarpal or acromioclavicular or sternoclavicular or glenoid or labral or labrum).ab,kf,ti. | 173,748 |
| 28 | (bankart* or SLAP lesion* or SLAP tear* or monteggia* or perilunate).ab,kf,ti. | 3,688 |
| 29 | (rotator cuff* or deltoid or pectoralis or biceps or triceps).ab,kf,ti. | 46,018 |
| 30 | bursitis.ab,kf,ti. | 3,253 |
| 31 | (brachial plexus or median nerve or musculocutaneous nerve).ab,kf,ti. | 22,567 |
| 32 | elbow*.ab,kf,ti. | 37,672 |
| 33 | adhesive capsulitis.ab,kf,ti. | 1,142 |
| 34 | (interphalangeal or inter phalangeal or metacarpophalangeal or carpometacarpal or metacarpophalangeal or radiocarpal or palmar plate* or volar plate* or triangular fibrocartilage).ab,kf,ti. | 15,660 |
| 35 | ("de quervain*" or dequervain* or trigger finger*).ab,kf,ti. | 1,801 |
| 36 | dupuytren*.ab,kf,ti. | 3,136 |
| 37 | (flexor* or extensor*).ab,kf,ti. | 51,096 |
| 38 | or/11-37 | 1,218,686 |
| 39 | 10 and 38 | **1,234** |

Updated Medline search 2^nd^ May 2023, 68 results.

**EMBASE**

**Database:**
Embase <1974 to 2022 May 10>

| **#** | **Query** | **Results from 11 May 2022** |
| --- | --- | --- |
| 1 | hydrotherapy/ or hydrotherapy equipment/ | 4,021 |
| 2 | balneotherapy/ | 3,994 |
| 3 | (hydrotherap* or balneotherapy or balneology or balneopathy).ab,kf,ti. | 3,218 |
| 4 | ((aqua* adj4 exercis*) or (aqua* adj4 therap*) or (aqua* adj4 physiotherap*) or (aqua* adj4 physical therap*) or (aqua* adj4 rehabilitat*) or (aqua* adj4 train*)).ab,kf,ti. | 1,776 |
| 5 | ((water* adj4 exercis*) or (water* adj4 therap*) or (water* adj4 physiotherap*) or (water* adj4 physical therap*) or (water* adj4 rehabilitat*) or (water* adj4 train*)).ab,kf,ti. | 6,174 |
| 6 | ((aqua* adj4 ai chi) or (aqua* adj4 aichi) or (aqua* adj4 tai chi) or (aqua* adj4 taichi) or (aqua* adj4 running) or (aqua* adj4 aerobic*) or (aqua* adj4 fitness)).ab,kf,ti. | 338 |
| 7 | ((water* adj4 ai chi) or (water* adj4 aichi) or (water* adj4 tai chi) or (water* adj4 taichi) or (water* adj4 shiatsu) or Halliwick* or Burdenko* or "Bad Ragaz*" or watershiatsu or watsu or deep water running or (water* adj4 aerobic*) or (water* adj4 fitness)).ab,kf,ti. | 1,391 |
| 8 | (pool therap* or whirlpool or water bath*).ab,kf,ti. | 7,487 |
| 9 | or/1-8 | 23,849 |
| 10 | upper limb/ | 18,907 |
| 11 | arm/ or elbow/ or forearm/ or wrist/ | 153,914 |
| 12 | shoulder/ or exp shoulder girdle/ | 64,823 |
| 13 | hand/ | 30,491 |
| 14 | finger/ or index finger/ or little finger/ or middle finger/ or ring finger/ or thumb/ | 42,948 |
| 15 | arm bone/ or exp humerus/ or exp radius/ or exp ulna/ | 33,401 |
| 16 | hand bone/ or exp carpal bone/ or finger phalanx/ or metacarpal bone/ | 15,312 |
| 17 | arm injury/ or elbow dislocation/ or elbow injury/ | 9,181 |
| 18 | shoulder fracture/ or shoulder injury/ or bankart lesion/ or exp proximal humerus fracture/ | 8,646 |
| 19 | exp elbow fracture/ or arm fracture/ | 5,173 |
| 20 | forearm fracture/ | 3,105 |
| 21 | radius fracture/ or exp distal radius fracture/ or exp proximal radius fracture/ or radius shaft fracture/ | 13,008 |
| 22 | ulna fracture/ or monteggia fracture/ or olecranon fracture/ | 4,574 |
| 23 | wrist fracture/ or capitate fracture/ or hamate fracture/ or lunate fracture/ or metacarpal bone fracture/ or pisiform fracture/ or scaphoid fracture/ or trapezium fracture/ or triquetrum fracture/ | 6,979 |
| 24 | hand fracture/ or finger fracture/ or metacarpal bone fracture/ or thumb fracture/ | 3,377 |
| 25 | rotator cuff injury/ or rotator cuff rupture/ or rotator cuff tear arthropathy/ or shoulder impingement syndrome/ | 13,932 |
| 26 | shoulder dislocation/ | 6,187 |
| 27 | frozen shoulder/ | 2,316 |
| 28 | elbow dislocation/ | 1,709 |
| 29 | epicondylitis/ or medial epicondylitis/ or tennis elbow/ | 4,142 |
| 30 | wrist dislocation/ | 923 |
| 31 | shoulder pain/ | 19,077 |
| 32 | arm pain/ or hand pain/ or wrist pain/ | 8,686 |
| 33 | shoulder muscle/ or deltoid muscle/ | 6,087 |
| 34 | exp rotator cuff/ | 10,296 |
| 35 | exp pectoral muscle/ | 8,262 |
| 36 | acromioclavicular joint/ or radioulnar joint/ or sternoclavicular joint/ | 6,197 |
| 37 | hand joint/ or carpal joint/ or carpometacarpal joint/ or finger joint/ or metacarpophalangeal joint/ | 13,637 |
| 38 | palmar plate/ or volar plate/ | 607 |
| 39 | triangular fibrocartilage/ | 771 |
| 40 | bursitis/ | 5,263 |
| 41 | brachial plexus/ or median nerve/ or musculocutaneous nerve/ or radial nerve/ or ulnar nerve/ | 32,009 |
| 42 | carpal tunnel syndrome/ | 16,580 |
| 43 | radial neuropathy/ | 423 |
| 44 | cubital tunnel syndrome/ | 3,011 |
| 45 | wrist injury/ | 4,398 |
| 46 | hand injury/ | 8,749 |
| 47 | exp finger injury/ | 6,698 |
| 48 | thumb injury/ or thumb dislocation/ | 1,028 |
| 49 | De Quervain tenosynovitis/ | 621 |
| 50 | trigger finger/ | 1,010 |
| 51 | Dupuytren contracture/ | 3,236 |
| 52 | (upper extremit* or upper limb* or shoulder* or arm or arms or axilla or forearm* or wrist* or hand or hands or finger* or forefinger* or thumb* or digit or digits).ab,kf,ti. | 1,310,057 |
| 53 | (humerus or humeral or radius or radial head or ulna or ulnar or ulnal or olecranon or clavicle or hamate or lunate or capitate or pisiform or scaphoid or trapezium or trapezoid or triquetrum or phalan* or metacarp* or carpal or carpus or scapula or acromion or coracoid or glenoid or glenohumeral or radioulnar or radiocarpal or acromioclavicular or sternoclavicular or glenoid or labral or labrum).ab,kf,ti. | 198,140 |
| 54 | (bankart* or SLAP lesion* or SLAP tear* or monteggia* or perilunate).ab,kf,ti. | 4,178 |
| 55 | (rotator cuff* or deltoid or pectoralis or biceps or triceps).ab,kf,ti. | 55,711 |
| 56 | bursitis.ab,kf,ti. | 3,900 |
| 57 | (brachial plexus or median nerve or musculocutaneous nerve).ab,kf,ti. | 28,671 |
| 58 | "elbow*".ab,kf,ti. | 48,300 |
| 59 | adhesive capsulitis.ab,kf,ti. | 1,501 |
| 60 | (interphalangeal or inter phalangeal or metacarpophalangeal or carpometacarpal or metacarpophalangeal or radiocarpal or palmar plate* or volar plate* or triangular fibrocartilage).ab,kf,ti. | 18,932 |
| 61 | ("de quervain*" or dequervain* or trigger finger*).ab,kf,ti. | 1,995 |
| 62 | (flexor* or extensor*).ab,kf,ti. | 61,066 |
| 63 | or/10-62 | 1,581,533 |
| 64 | 9 and 63 | **1,979** |

Updated Embase search 2^nd^ May 2023, 138 results.

**CINAHL**

| **#** | **Query** | **Results** |
| --- | --- | --- |
| S1 | (MH "Hydrotherapy") | 1,686 |
| S2 | (MH "Aquatic Exercises") | 1,382 |
| S3 | (MH "Balneology") | 643 |
| S4 | TI ( hydrotherap* or balneotherapy or balneology or balneopathy ) OR AB ( hydrotherap* or balneotherapy or balneology or balneopathy ) OR SU ( hydrotherap* or balneotherapy or balneology or balneopathy ) | 2,546 |
| S5 | TI ( (aqua* N4 exercis*) or (aqua* N4 therap*) or (aqua* N4 physiotherap*) or (aqua* N4 "physical therap*") or (aqua* N4 rehabilitat*) or (aqua* N4 train*) ) OR AB ( (aqua* N4 exercis*) or (aqua* N4 therap*) or (aqua* N4 physiotherap*) or (aqua* N4 "physical therap*") or (aqua* N4 rehabilitat*) or (aqua* N4 train*) ) OR SU ( (aqua* N4 exercis*) or (aqua* N4 therap*) or (aqua* N4 physiotherap*) or (aqua* N4 "physical therap*") or (aqua* N4 rehabilitat*) or (aqua* N4 train*) ) | 1,962 |
| S6 | TI ( (water* N4 exercis*) or (water* N4 therap*) or (water* N4 physiotherap*) or (water* N4 "physical therap*") or (water* N4 rehabilitat*) or (water* N4 train*) ) OR AB ( (water* N4 exercis*) or (water* N4 therap*) or (water* N4 physiotherap*) or (water* N4 "physical therap*") or (water* N4 rehabilitat*) or (water* N4 train*) ) OR SU ( (water* N4 exercis*) or (water* N4 therap*) or (water* N4 physiotherap*) or (water* N4 "physical therap*") or (water* N4 rehabilitat*) or (water* N4 train*) ) | 1,953 |
| S7 | TI ( (aqua* N4 "ai chi") or (aqua* N4 aichi) or (aqua* N4 "tai chi") or (aqua* N4 taichi) or (aqua* N4 running) or (aqua* N4 aerobic*) or (aqua* N4 fitness) ) OR AB ( (aqua* N4 "ai chi") or (aqua* N4 aichi) or (aqua* N4 "tai chi") or (aqua* N4 taichi) or (aqua* N4 running) or (aqua* N4 aerobic*) or (aqua* N4 fitness) ) OR SU ( (aqua* N4 ai chi) or (aqua* N4 aichi) or (aqua* N4 tai chi) or (aqua* N4 taichi) or (aqua* N4 running) or (aqua* N4 aerobic*) or (aqua* N4 fitness) ) | 147 |
| S8 | TI ( (water* N4 "ai chi") or (water* N4 aichi) or (water* N4 "tai chi") or (water* N4 taichi) or (water* N4 shiatsu) or Halliwick* or Burdenko* or "Bad Ragaz*" or watershiatsu or watsu or "deep water running" or (water* N4 aerobic*) or (water* N4 fitness) ) OR AB ( (water* N4 "ai chi") or (water* N4 aichi) or (water* N4 "tai chi") or (water* N4 taichi) or (water* N4 shiatsu) or Halliwick* or Burdenko* or "Bad Ragaz*" or watershiatsu or watsu or "deep water running" or (water* N4 aerobic*) or (water* N4 fitness) ) OR SU ( (water* N4 "ai chi") or (water* N4 aichi) or (water* N4 "tai chi") or (water* N4 taichi) or (water* N4 shiatsu) or Halliwick* or Burdenko* or "Bad Ragaz*" or watershiatsu or watsu or "deep water running" or (water* N4 aerobic*) or (water* N4 fitness) ) | 325 |
| S9 | TI ( "pool therap*" or whirlpool or "water bath*" ) OR AB ( "pool therap*" or whirlpool or "water bath*" ) OR SU ( "pool therap*" or whirlpool or "water bath*" ) | 682 |
| S10 | S1 OR S2 OR S3 OR S4 OR S5 OR S6 OR S7 OR S8 OR S9 | 6,168 |
| S11 | (MH "Upper Extremity+") | 44,381 |
| S12 | (MH "Arm Bones") | 95 |
| S13 | (MH "Arm Injuries+") | 18,132 |
| S14 | (MH "Shoulder Pain") OR (MH "Elbow Pain") | 4,615 |
| S15 | (MH "Deltoid Muscles") OR (MH "Pectoralis Muscles") OR (MH "Rotator Cuff+") OR (MH "Triceps Brachii") OR (MH "Flexor Pollicis Longus Muscle") OR (MH "Flexor Hallucis Longus") OR (MH "Biceps Brachii Muscles") | 5,828 |
| S16 | (MH "Acromioclavicular Joint") OR (MH "Elbow Joint") OR (MH "Hand Joints+") OR (MH "Shoulder Joint+") | 13,791 |
| S17 | (MH "Bursitis") | 1,050 |
| S18 | (MH "Adhesive Capsulitis+") | 744 |
| S19 | (MH "Brachial Plexus+") | 4,875 |
| S20 | (MH "Brachial Plexus Neuropathies+") OR (MH "Carpal Tunnel Syndrome") OR (MH "Ulnar Nerve Compression Syndromes+") | 4,774 |
| S21 | (MH "De Quervain Disease") OR (MH "Trigger Finger Disorder") | 403 |
| S22 | (MH "Dupuytren's Contracture") | 410 |
| S23 | AB ( "upper extremit*" or "upper limb*" or shoulder* or arm or arms or axilla or forearm* or wrist* or hand or hands or finger* or forefinger* or thumb* or digit or digits ) OR TI ( "upper extremit*" or "upper limb*" or shoulder* or arm or arms or axilla or forearm* or wrist* or hand or hands or finger* or forefinger* or thumb* or digit or digits ) OR SU ( "upper extremit*" or "upper limb*" or shoulder* or arm or arms or axilla or forearm* or wrist* or hand or hands or finger* or forefinger* or thumb* or digit or digits ) | 223,901 |
| S24 | TI ( humerus or humeral or radius or "radial head" or ulna or ulnar or ulnal or olecranon or clavicle or hamate or lunate or capitate or pisiform or scaphoid or trapezium or trapezoid or triquetrum or phalan* or metacarp* or carpal or carpus or scapula or acromion or coracoid or glenoid or glenohumeral or radioulnar or radiocarpal or acromioclavicular or sternoclavicular or glenoid or labral or labrum ) OR AB ( humerus or humeral or radius or "radial head" or ulna or ulnar or ulnal or olecranon or clavicle or hamate or lunate or capitate or pisiform or scaphoid or trapezium or trapezoid or triquetrum or phalan* or metacarp* or carpal or carpus or scapula or acromion or coracoid or glenoid or glenohumeral or radioulnar or radiocarpal or acromioclavicular or sternoclavicular or glenoid or labral or labrum ) OR SU ( humerus or humeral or radius or "radial head" or ulna or ulnar or ulnal or olecranon or clavicle or hamate or lunate or capitate or pisiform or scaphoid or trapezium or trapezoid or triquetrum or phalan* or metacarp* or carpal or carpus or scapula or acromion or coracoid or glenoid or glenohumeral or radioulnar or radiocarpal or acromioclavicular or sternoclavicular or glenoid or labral or labrum ) | 40,131 |
| S25 | TI ( bankart* or "SLAP lesion*" or "SLAP tear" or monteggia* or perilunate ) OR AB ( bankart* or "SLAP lesion*" or "SLAP tear" or monteggia* or perilunate ) OR SU ( bankart* or "SLAP lesion*" or "SLAP tear" or monteggia* or perilunate ) | 1,414 |
| S26 | TI ( "rotator cuff*" or deltoid or pectoralis or biceps or triceps ) OR AB ( "rotator cuff*" or deltoid or pectoralis or biceps or triceps ) OR SU ( "rotator cuff*" or deltoid or pectoralis or biceps or triceps ) | 16,435 |
| S27 | TI bursitis OR AB bursitis OR SU bursitis | 1,565 |
| S28 | TI ( "brachial plexus" or "median nerve" or "musculocutaneous nerve" ) OR AB ( "brachial plexus" or "median nerve" or "musculocutaneous nerve" ) OR SU ( "brachial plexus" or "median nerve" or "musculocutaneous nerve" ) | 6,710 |
| S29 | TI elbow* OR AB elbow* OR SU elbow* | 14,091 |
| S30 | TI "adhesive capsulitis" OR AB "adhesive capsulitis" OR SU "adhesive capsulitis" | 1,042 |
| S31 | TI ( interphalangeal or "inter phalangeal" or metacarpophalangeal or carpometacarpal or metacarpophalangeal or radiocarpal or "palmar plate*" or "volar plate*" or "triangular fibrocartilage" ) OR AB ( interphalangeal or "inter phalangeal" or metacarpophalangeal or carpometacarpal or metacarpophalangeal or radiocarpal or "palmar plate*" or "volar plate*" or "triangular fibrocartilage" ) OR SU ( interphalangeal or "inter phalangeal" or metacarpophalangeal or carpometacarpal or metacarpophalangeal or radiocarpal or "palmar plate*" or "volar plate*" or "triangular fibrocartilage" ) | 3,472 |
| S32 | TI ( "de quervain*" or dequervain* or "trigger finger*" ) OR AB ( "de quervain*" or dequervain* or "trigger finger*" ) OR SU ( "de quervain*" or dequervain* or "trigger finger*" ) | 584 |
| S33 | TI dupuytren* OR AB dupuytren* OR SU dupuytren* | 517 |
| S34 | TI ( flexor* or extensor* ) OR AB ( flexor* or extensor* ) OR SU ( flexor* or extensor* ) | 13,268 |
| S35 | S11 OR S12 OR S13 OR S14 OR S15 OR S16 OR S17 OR S18 OR S19 OR S20 OR S21 OR S22 OR S23 OR S24 OR S25 OR S26 OR S27 OR S28 OR S29 OR S30 OR S31 OR S32 OR S33 OR S34 | 263,669 |
| S36 | S10 AND S35 | **561** |

Updated CINAHL search 2^nd^ May 2023, 55 results.

**CENTRAL**

#1 MeSH descriptor: [Hydrotherapy] this term only 214

#2 MeSH descriptor: [Aquatic Therapy] this term only 1

#3 MeSH descriptor: [Balneology] this term only 187

#4 (hydrotherap* or balneotherapy or balneology or balneopathy):ti,ab,kw 974

#5 ((aqua* NEAR/4 exercis*) or (aqua* NEAR/5 therap*) or (aqua* NEAR/4 physiotherap*) or (aqua* NEAR/4 rehabilitat*) or (aqua* NEAR/4 train*)):ti,ab,kw 785

#6 ((water NEAR/4 exercis*) or (water NEAR/5 therap*) or (water NEAR/4 physiotherap*) or (water NEAR/4 rehabilitat*) or (water NEAR/4 train*)):ti,ab,kw 1622

#7 ((aqua* NEAR/4 ai) or (aqua* NEAR/4 aichi) or (aqua* NEAR/4 tai) or (aqua* NEAR/4 taichi) or (aqua* NEAR/4 running) or (aqua* NEAR/4 aerobic*) or (aqua* NEAR/4 fitness)):ti,ab,kw 129

#8 ((water* NEAR/4 ai) or (water* NEAR/4 aichi) or (water* NEAR/4 tai) or (water* NEAR/4 taichi) or (water* NEAR/4 shiatsu) or Halliwick* or Burdenko* or "Bad Ragaz" or watershiatsu or watsu or "deep water running" or (water* NEAR/4 aerobic*) or (water* NEAR/4 fitness)):ti,ab,kw 191

#9 ("pool therapy" or "pool therapies" or whirlpool or "water bath" or "water baths"):ti,ab,kw 325

#10 {OR #1-#9} 3249

#11 MeSH descriptor: [Upper Extremity] explode all trees 7971

#12 MeSH descriptor: [Bones of Upper Extremity] explode all trees 834

#13 MeSH descriptor: [Shoulder Injuries] explode all trees 1311

#14 MeSH descriptor: [Monteggia's Fracture] explode all trees 1

#15 MeSH descriptor: [Colles' Fracture] this term only 110

#16 MeSH descriptor: [Shoulder Pain] this term only 1093

#17 MeSH descriptor: [Deltoid Muscle] explode all trees 24

#18 MeSH descriptor: [Pectoralis Muscles] explode all trees 80

#19 MeSH descriptor: [Rotator Cuff] explode all trees 436

#20 MeSH descriptor: [Acromioclavicular Joint] this term only 59

#21 MeSH descriptor: [Elbow Joint] this term only 274

#22 MeSH descriptor: [Hand Joints] explode all trees 583

#23 MeSH descriptor: [Shoulder Joint] this term only 816

#24 MeSH descriptor: [Bursitis] this term only 380

#25 MeSH descriptor: [Brachial Plexus] explode all trees 1019

#26 MeSH descriptor: [Elbow Tendinopathy] this term only 9

#27 MeSH descriptor: [Median Neuropathy] explode all trees 780

#28 MeSH descriptor: [Radial Neuropathy] this term only 6

#29 MeSH descriptor: [Ulnar Neuropathies] explode all trees 63

#30 MeSH descriptor: [Hand Injuries] explode all trees 306

#31 MeSH descriptor: [De Quervain Disease] explode all trees 32

#32 MeSH descriptor: [Trigger Finger Disorder] this term only 106

#33 MeSH descriptor: [Dupuytren Contracture] this term only 103

#34 ("upper extremity" or "upper extremities" or "upper limb" or "upper limbs" or shoulder* or arm or arms or axilla or forearm* or wrist* or hand or hands or finger* or forefinger* or thumb* or digit or digits):ti,ab,kw 196163

#35 (humerus or humeral or radius or "radial head" or ulna or ulnar or ulnal or olecranon or clavicle or hamate or lunate or capitate or pisiform or scaphoid or trapezium or trapezoid or triquetrum or phalan* or metacarp* or carpal or carpus or scapula or acromion or coracoid or glenoid or glenohumeral or radioulnar or radiocarpal or acromioclavicular or sternoclavicular or glenoid or labral or labrum):ti,ab,kw 11249

#36 (bankart* or "SLAP lesion" or "SLAP lesions" or "SLAP tear" or "SLAP tears" or monteggia* or perilunate):ti,ab,kw 185

#37 ("rotator cuff" or "rotator cuffs" or deltoid or pectoralis or biceps or triceps):ti,ab,kw 6045

#38 (bursitis):ti,ab,kw 616

#39 ("brachial plexus" or "median nerve" or "musculocutaneous nerve"):ti,ab,kw 3434

#40 (elbow*):ti,ab,kw 4975

#41 ("adhesive capsulitis"):ti,ab,kw 461

#42 (interphalangeal or "inter phalangeal" or metacarpophalangeal or carpometacarpal or metacarpophalangeal or radiocarpal or "palmar plate" or "palmar plates" or "volar plate" or "volar plates" or "triangular fibrocartilage"):ti,ab,kw 1186

#43 ("de quervain" or "de quervains" or "de quervain's" or "trigger finger" or "trigger fingers"):ti,ab,kw 306

#44 (dupuytren*):ti,ab,kw 192

#45 (flexor* or extensor*):ti,ab,kw 6306

#46 {OR #11-#45} 208569

#47 #10 AND #46 in Trials **625**

Updated Central search 2^nd^ May 2023, 65 results.

**EThOS search strategy – advanced search**

**Searches carried out: 12/5/22**

**Carried out on advanced search**

| **Search term** | **Search criteria** | **Number of records from search** | **Number of relevant records** |
| --- | --- | --- | --- |
| hydrotherapy | Any word | 9 records | 0 |
| aquatic therapy | Any word | 1 record | 0 |
| balneology | Any word | 2 records | 0 |
| balneopathy | Any word | 0 records | 0 |
| balneotherapy | Any word | 2 records | 0 |
| aquatic physiotherapy | Any word | 2 records | 0 |
| aquatic exercise | Any word | 0 records | 0 |
| aquatic training | Any word | 0 records | 0 |
| aquatic physical therapy | Any word | 0 records | 0 |
| aquatic rehabilitation | Any word | 0 records | 0 |
| water training | Any word | 6 records | 0 |
| water rehabilitation | Any word | 0 records | 0 |
| water physical therapy | Any word | 0 records | 0 |
| water physiotherapy | Any word | 0 records | 0 |
| water therapy | Any word | 0 records | 0 |
| water exercise | Any word | 2 records | 0 |
| water tai chi | Any word | 0 records | 0 |
| ai chi | Any word | 0 records | 0 |
| aqua aerobics | Any word | 1 record | 0 |
| aqua fitness | Any word | 0 records | 0 |
| Halliwick | Any word | 0 records | 0 |
| Burdenko | Any word | 0 records | 0 |
| Bad Regaz | Any word | 0 records | 0 |
| water shiatsu | Any word | 0 records | 0 |
| Watsu | Any word | 0 records | 0 |
| deep water running | Any word | 1 record | 0 |
| whirlpool | Any word | 11 records | 0 |
| water bath | Abstract | 56 records | 0 |

**Outcome:** 0 relevant articles found

**NDLTD Global ETD Search**

**Searches carried out 12/5/22**

| **Refine Query** | **Number of search results** | **Number of relevant results** |
| --- | --- | --- |
| hydrotherapy | 136 | 1 |
| “aquatic therapy” | 29 | 1 |
| balneology | 40 | 0 |
| balneopathy | 0 | 0 |
| balneotherapy | 23 | 0 |
| “aquatic physiotherapy” | 10 | 0 |
| “aquatic exercise” | 75 | 0 |
| “aquatic training” | 16 | 0 |
| “aquatic physical therapy” | 6 | 0 |
| “aquatic rehabilitation” | 7 | 0 |
| “water training” | 23 | 0 |
| “water rehabilitation” | 2 | 0 |
| “water physical therapy” | 1 | 0 |
| “water physiotherapy” | 0 | 0 |
| “water therapy” | 14 | 0 |
| “water exercise” | 44 | 1 |
| “water tai chi” | 0 | 0 |
| “ai chi” | 406 – reduce using terms for upper extremity (see below) |  |
| “ai chi” AND “upper extremity” | 1 | 0 |
| “ai chi” AND arm | 2 | 0 |
| “ai chi” AND shoulder | 13 | 0 |
| “ai chi” AND elbow | 0 | 0 |
| “ai chi” AND wrist | 0 | 0 |
| “ai chi” AND hand | 20 | 0 |
| “ai chi” AND finger | 0 | 0 |
| “ai chi” AND thumb | 0 | 0 |
| “aqua aerobics” | 9 | 0 |
| “aqua fitness” | 4 | 0 |
| Halliwick | 7 | 0 |
| Burdenko | 36 | 0 |
| “Bad Regaz” | 0 | 0 |
| “water shiatsu” | 0 | 0 |
| Watsu | 7 | 0 |
| “deep water running” | 20 | 0 |
| whirlpool | 153 | 0 |
| “water bath” | 846 – reduce using terms for upper extremity |  |
| “water bath” AND “upper extremity” | 0 | 0 |
| “water bath” AND arm | 6 | 0 |
| “water bath” AND shoulder | 4 | 0 |
| “water bath” AND elbow | 0 | 0 |
| “water bath” AND wrist | 0 | 0 |
| “water bath” AND hand | 50 | 0 |
| “water bath” AND finger | 3 | 0 |
| “water bath” AND thumb | 0 | 0 |

**Outcome:** 3 relevant articles found

**Google Advanced search**

**Searches carried out: 12/5/2022**

In each search:

Results were limited to PDF files.

The first 200 results only were examined.

| **All these words** | **AND ANY of these words** | **Number of relevant results** |
| --- | --- | --- |
| "upper limb" | hydrotherapy "aquatic physiotherapy" "aquatic therapy" "physical therapy" "aquatic exercise" aquatherapy | 2 |
| "upper extremity" | hydrotherapy "aquatic physiotherapy" "aquatic therapy" "physical therapy" "aquatic exercise" aquatherapy | 2 |
| “rotator cuff” | hydrotherapy "aquatic physiotherapy" "aquatic therapy" "physical therapy" "aquatic exercise" aquatherapy | 3 |
| elbow | hydrotherapy "aquatic physiotherapy" "aquatic therapy" "physical therapy" "aquatic exercise" aquatherapy | 0 |
| arm | hydrotherapy "aquatic physiotherapy" "aquatic therapy" "physical therapy" "aquatic exercise" aquatherapy | 1 |
| wrist | hydrotherapy "aquatic physiotherapy" "aquatic therapy" "physical therapy" "aquatic exercise" aquatherapy | 0 |
| hand | hydrotherapy "aquatic physiotherapy" "aquatic therapy" "physical therapy" "aquatic exercise" aquatherapy | 0 |

**Outcome:** 8 relevant items found

**Grey literature - Special Interest Group Contact**

Contact with aquatic special interest groups across the world was decided upon by looking at the World Physiotherapy Subgroup, The International Organisation of Aquatic Physical Therapists (IOAPT).

The member organisations are:

- Asociación Argentina de Hidroterapia de la Asociación Argentina de kinesiologia (AAK) (Argentina)
- Aquatic Physiotherapy Group of the Australian Physiotherapy Association (APA) (Australia)
- Associação Brasileira de Fisioterapia Aquática (ABFA) (Brazil)
- Danish Aquatic Physiotherapy Group (Denmark)
- Asociación Mexicana de Fisioterapia (AMEFI) (Mexico)
- Grupo de Interesse em Fisioterapia Aquática - Hidroterapia (GIFA) of the Associação Portuguesa de Fisioterapeutas (APFISIO) (Portugal)
- Aquatic Physiotherapy Group (AQPG) (South Africa)
- Grupo de Interés de Fisioterapia en el Medio Acuático of the Asociación Española de  Fisioterapeutas (AEF) (Spain)
- Aquatic Therapy Association of Chartered Physiotherapists (ATACP) (United Kingdom)
- Academy of Aquatic Physical Therapy (United States)

In line with the scoping review protocol, we have limited the setting to any highly developed nation (defined as the top 66 countries in the Human Development Index).

This left the:

- Asociación Argentina de Hidroterapia de la Asociación Argentina de kinesiologia (AAK) (Argentina)
- Aquatic Physiotherapy Group of the Australian Physiotherapy Association (APA) (Australia)
- Danish Aquatic Physiotherapy Group (Denmark)
- Grupo de Interesse em Fisioterapia Aquática - Hidroterapia (GIFA) of the Associação Portuguesa de Fisioterapeutas (APFISIO) (Portugal)
- Grupo de Interés de Fisioterapia en el Medio Acuático of the Asociación Española de Fisioterapeutas (AEF) (Spain)
- Aquatic Therapy Association of Chartered Physiotherapists (ATACP) (United Kingdom)
- Academy of Aquatic Physical Therapy (United States)
- No contact was gained for the Argentinian, Danish or Spanish associations despite 2 attempts.
- Online meetings were held with contacts provided from the Australian & Portuguese associations.
- No articles gained from the Australian contact but 4 articles gained from the Portuguese contact.
- Online access to the USA Academy of Aquatic Physical Therapy Journal. Searches run 2/6/22.
- Online access to the UK Aqualines Journal of the Aquatic Therapy Association of Chartered Physiotherapists – articles hand searched from Aqualines Spring 1999 to the last published journal in September 2020. Searches run 5/5/22.

**Grey Literature Searches of The Journal of Aquatic Physical Therapy (USA)**

**Advanced search builder 26/5/22**

**Articles, podcasts, videos, blogs**

**All date**

**All article types**

| **Keyword**  **All fields** | **Search Results** | **Number of relevant articles** | **Number of articles once duplicates removed** |
| --- | --- | --- | --- |
| Shoulder | 28 | 6 | 6 |
| "upper extremity” | 21 | 2 (1 duplicate) | 1 |
| arm | 29 | 2 (2 duplicate) | 0 |
| elbow | 4 | 0 | 0 |
| forearm | 2 | 0 | 0 |
| wrist | 6 | 0 | 0 |
| finger | 5 | 0 | 0 |
| thumb | 0 | 0 | 0 |
| hand | 41 | 2 (1 duplicate) | 1 |
| humerus | 0 | 0 | 0 |
| ulna | 0 | 0 | 0 |
| radius | 0 | 0 | 0 |
| Metacarpal | 0 | 0 | 0 |
| Carpal | 3 | 0 | 0 |
| phalanx | 0 | 0 | 0 |
| phalanges | 0 | 0 | 0 |
| glenohumeral | 1 | 1 | 0 |
| acromioclavicular | 0 | 0 | 0 |
| fracture | 6 | 0 | 0 |
| dislocation | 2 | 0 | 0 |
| tendinopathy | 2 | 0 | 0 |
| bursitis | 0 | 0 | 0 |
| “brachial plexus” | 0 | 0 | 0 |
| “peripheral nerve injury” | 0 | 0 | 0 |
| **TOTAL** | **150** | **12** | **8** |

**Final totals gained from Special Interest Groups**

| **Country** | **Any articles gained** |
| --- | --- |
| Argentina | 0 |
| Australia | 0 |
| Denmark | 0 |
| Portugal | 4 |
| Spain | 0 |
| UK | 8 |
| USA | 8 |

**Outcome**: 20 relevant sources found
