# Supplementary material for: Aquatic exercise interventions in the treatment of musculoskeletal upper extremity disorders: A scoping review
Source: Clin Rehabil. 2025 Feb 2;39(5):565–79. doi: 10.1177/02692155251315078 (PMC12099020; doi:10.1177/02692155251315078)
Supplement: sj-doc-2-cre-10.1177_02692155251315078 - Supplemental material for Aquatic exercise interventions in the treatment of musculoskeletal upper extremity disorders: A scoping review [file sj-doc-2-cre-10.1177_02692155251315078.doc]

| **Reason for Exclusion** | **Citation** |
| --- | --- |
| **Duplicate n=8** | |
| Duplicate | Babb R. A Role for Hydrotherapy in Shoulder Rehabilitation. Journal of Aquatic Physical Therapy 1996;4(3):18. |
| Duplicate | Cikes A, Kadri F, Ladermann A. Evaluation of three different rehabilitation protocols after rotator cuff repair, and the effectiveness of water/pool therapy. A randomized control study. Swiss Med Wkly 2020;150 (Supp 244):4s. |
| Duplicate | ClinicalTrials.gov. [Internet] Bethesda (MD): National Library of Medicine (US). 2000 Feb 29 -. Identifier NCT05106842, Hydrotherapy Versus Classical Rehabilitation After Surgical Rotator Cuff Repair; 2021 Nov 4 [cited 2023 April 05] [about 6 screens]. Available from: <https://clinicaltrials.gov/ct2/show/study/NCT05106842> |
| Duplicate | Daly AE, & Bialocerkowski AE. Does evidence support physiotherapy management of adult Complex Regional Pain Syndrome Type One? A systematic review. Eur J Pain 2008;13(4):339-353. |
| Duplicate | Kearney D, Lumia R, Siegel R, Szemenyei, S, Leininger. Effectiveness of aquatic therapy on increasing range of motion and decreasing pain in the rehabilitation of patients with shoulder pathologies: a systematic review [PowerPoint presentation]. University of Scranton. [2017 July 11**;** cited 2023 April 6]. Available from: <https://www.scranton.edu/academics/pcps/physicaltherapy/ptresearch/DPT-3-PowerPoints-2017/at-for-rotator-cuff-repair-and-shoulder-impingement-group-7.pdf> |
| Duplicate | Laedermann A, Kadri F, Cikes A. Evaluation of 3 different rehabilitation protocols after rotator cuff repair, and the effectiveness of pool therapy: a randomized control study. Orthop J Sports Med 2021;9(2 Supp):2325967121. |
| Duplicate | Tyler TF, Nicholas SJ, Schmitt BM, Mullaney M, Hogan DE. Clinical outcomes of the addition of eccentrics for rehabilitation of previously failed treatments of golfers elbow. Int J Sports Phys Ther 2014;9(3)365-370. |
| Duplicate | Laedermann A, Cikes A, Zbinden J, Martinho T, Pernoud A, Bothorel H. Hydrotherapy after Rotator Cuff Repair Improves Short-Term Functional Results Compared with Land-Based Rehabilitation When the Immobilization Period Is Longer. Journal of Clinical Medicine. 2024; 13(4):954. |
| **Not on HDI very high 2022 list n=5** | |
| Not on HDI very high 2022 list | Iranian Registry of Clinical Trials [Internet]: Tehran: IRCT, Central Library Building, Iran University Campus University. 2008 -. Identifier, IRCT20200511047403N1. Effects of hydrotherapy versus land based exercises on pain and grip strength in osteoarthritis of hand; 2020 June 8 [cited 2023 April 06] [about 11 screens]. Available from: <https://clinicaltrials.gov/ct2/show/study/NCT05106842> |
| Not on HDI very high 2022 list | Odynets T, Briskin Y, Sydorko O, et al. Effectiveness of individualized physical rehabilitation programs on post-mastectomy pain in breast cancer survivors. Physiotherapy Quarterly 2018;26(3):1-5. |
| Not on HDI very high 2022 list | Odynets T, Briskin Y, Perederiy A, Pityn M, Svistelnyk I. Effect of water physical therapy on quality of life in breast cancer survivors. Physiotherapy Quarterly 2018;26(4):11-16. |
| Not on HDI very high 2022 list | Siqueira RB, Freitas-Junior R, Lopes PS, Lagares MS, Viana FP, Mesquita TM, Soares LR. Hydrotherapy following breast cancer surgery Phase II trial on hydrotherapy in women following breast cancer surgery. Breast J 2020;26(5):1107-1110. |
| Not on HDI very high 2022 list | Saul M.; Battistella L.R.; Bazan M.; Brito C.M.M.; Cecatto R.B.; Imamura M.; Lourencao M.I.P.; Otsubo P.S. PM and R / 2012;4(10 SUPPL. 1):S211-S212 |
| **Unable to access translation via Google translate n=2** | |
| Unable to access translation via Google translate | Hrncar I. Ergodiagnostics for requirements of physiatry, balneology and treatment rehabilitation. Rehabilitacia 2002;35(4):241-249. |
| Unable to access translation via Google translate | Livain T, Vial B, Charpin C, Lacombe P, Pigeon E. Roi M. Rehabilitation of patients with algoneurodystrophies of the hand and arm. Cahiers de Kinesitherapie 1988;130(2):67-79. |
| **Unable to extract relevant data n=19** | |
| Unable to extract relevant data | Bender T, Bálint G, Géher P, Prohászka Z, Tefner IK. PT2-1 EBM Balneotherapy in Hungary. Journal of the Japanese Society of Balneology, Climatology & Physical Medicine 2014;77(5):392. |
| Unable to extract relevant data | Bruder A,Taylor N, Dodd K, Shields N. Physiotherapy for the rehabilitation of upper limb fractures in adults: a systematic review and meta-analysis. Physiotherapy 2011;97(Supp 1):eS163. |
| Unable to extract relevant data | Curboy RA. The effects of participation in an arthritis foundation sponsored aquacize program on health status (Master of Science in Occupational Therapy Thesis): Miami, Florida: Florida International University; 1999. |
| Unable to extract relevant data | Durrant L, Beasley C, House S, Argles J, Batchelor A, Dobie F. Take the plunge…swimming rehabilitation to support recovery and promote self management in breast cancer patients. Support Care Cancer 2021;29(Supp 1):S95. |
| Unable to extract relevant data | Enblom A, Wicher M, Nordell T. Health-related quality of life and musculoskeletal function in patients with musculoskeletal disorders: after compared to before short-term group-based aqua-exercises. Eur J of Physiother 2016;18(4):218-225. |
| Unable to extract relevant data | Gangaway JM. Evidence in practice: use of aquatic physical therapy for an adult following clavicle resection. Journal of Aquatic Physical Therapy 2005;13(1):13-20. |
| Unable to extract relevant data | Gleyze P, Georges T, Flurin PH, et al. Comparison and critical evaluation of rehabilitation and home-based exercises for treating shoulder stiffness: prospective, multicenter study with 148 cases. Orthop Traumatol Surg Res 2011;97(8 Supp):S182-S194. |
| Unable to extract relevant data | Hirasawa Y, Yamaguchi Y, Okajima S. Surgical approach to cubital tunnel syndrome in the symptomatic osteoarthritic elbow. Orthopedics and Traumatology 2002;10(2):130-137. |
| Unable to extract relevant data | Hay L, Ahmed N. BET 4: Hydrotherapy following rotator cuff repair. Emerg Med J 2011;28(7):634-635. |
| Unable to extract relevant data | Norton CO, Hoobler K, Welding AB, et al. Effectiveness of aquatic exercise in the treatment of women with osteoarthritis. Journal of Aquatic Physical Therapy 1997;5(3):8-15. |
| Unable to extract relevant data | Roques C, Mancret RC, Tabone, W. Beneficial effect of balneotherapy: achievements of the French association of balneotherapy research and methodological difficulties. Ann Phys Rehabil Med 2012;55(Supp 1):e349. |
| Unable to extract relevant data | Salazar-Mendez J.; Morales-Verdugo J.; Lluch-Girbes E.; Araya-Quintanilla F.; Ribeiro I.L.; Mendoza-Grau R.; Mellado-Mendez J.; Mendez-Rebolledo G. A scoping review of tendon transfer surgeries and postsurgical physical therapy interventions in individuals with massive rotator cuff tears. PM and R / 2024;16(7):745-765 |
| Unable to extract relevant data | Sayliss, L. Review of ‘Aquatic therapy foundation principles (2009). Aqualines: the news magazine/journal of the Hydrotherapy Association of Physiotherapists 2011;23(2);23-24. |
| Unable to extract relevant data | Scott, PJ. Musculoskeletal injury outcomes: 2-year retrospective service evaluation of a UK defence primary care rehabilitation facility (PCRF). BMJ Mil Health 2021;167(3):182-186. |
| Unable to extract relevant data | Singh G, Willen S, Boswell M, Janata J, Chelimsky, T. The value of interdisciplinary pain management in complex regional pain syndrome type I: a prospective outcome study. Pain Physician 2004;7(2):203-209. |
| Unable to extract relevant data | Stover, A, Smith C, Maloney J, Mitchell C, Michaels, N, Jones T, Raynes, E. Aquatic exercise for pain reduction in the active adult with osteoarthritis. Journal of Aquatic Physical Therapy 2015;23(2):13-19. |
| Unable to extract relevant data | Stridh, K. Bassängträning, patientupplevelser och mål.: Hydrotherapy, patients´ experiences and goals (Bachelor degree dissertation): Örebro, Sweden. Örebro Universitet; 2007**.** |
| Unable to extract relevant data | Wratten, S. A Service evaluation of the use of Ai Chi as part of the rehabilitation program for UK military patients. 5th International Conference For Evidence Based Aquatic Therapy (ICEBAT), Apr 14-16, 2018, Las Vegas. Journal of Aquatic Physical Therapy 2018;26(2):23-24. |
| **Unable to source publication n=27** | |
| Unable to source publication | Ayrolles, C. [Clinical investigation on the action of luchon sodium-sulfurated waters on degenerative rheumatism (coxarthrosis and scapulo-humeral periarthritis)]. Presse Therm Clim 1964;101:35-39. |
| Unable to source publication | Bicilioglu C, El O, Kizil R Pehlivan K, Sagol A, Soylev G, Baydar M. Comparison of the therapeutic approaches of the patients with lateral epicondylitis: splint versus splint and physical therapy. Journal of Rheumatology and Medical Rehabilitation 2009;20(4):120-125. |
| Unable to source publication | Boileau P, Caligaris-Cordero B, Payeur F, et al. Prognostic factors after rehabilitation following shoulder arthroplasty for fracture. Rev Chir Orthop Reparatrice Appar Mot 1999;85(2):106-116. |
| Unable to source publication | Chiu S, Babatunde O, Corp N, Forsyth J, Van der Windt D, Paskins Z. Systematic review of patient outcomes and treatment moderators following wrist fractures. Br J Surg 2019;106(Supp 6):101. |
| Unable to source publication | Clark AJ. Comparison of aquatic and land intervention on arthroscopic subacromial decompression and distal clavicle resection...Poster presentations at APTA Combined Sections Meeting 2009.  Journal of Aquatic Physical Therapy Spring 2009;17(1):22. |
| Unable to source publication | Crevoisier J, Poindessous JL. Balneology. Soins 2008;(726):69-70. |
| Unable to source publication | De Ruvo F.; Zanazzo M. Efficacy of hydrotherapy on patients underwent a rotator cuff surgical repair. Gazz Med Ital 2014;173(11):539-545. |
| Unable to source publication | Dockrell S, Wiseman J. Current physiotherapy treatment of frozen shoulder. BJTR pull-out physiotherapy supplement on rheumatology. British Journal of Therapy & Rehabilitation 1995;2(4):189-194. |
| Unable to source publication | Emrich M. Possibilities and methods of balneophysiotherapy and medical recuperation in scapulo-humeral periarthritis. Viata Med Rev Inf Prof Stiint Cadrelor Medii Sanit 1979;27(7):155-156. |
| Unable to source publication | Faubel W. Rehabilitation of persons with limb injuries. Hefte Unfallheilkd 1974;(117):186-190. |
| Unable to source publication | Garabini MC, Leite CM, Borba,G. The use of the water Pilates method in the occupationally originated painful syndrome in the upper limbs. Revista Terapia Manual 2011;9(41):63-68. |
| Unable to source publication | Gouhot, P. [Shoulder pain and the spa of Bourbon l'Archambault]. Concours Med 1954;76(12):1173-1175. |
| Unable to source publication | Kotwica S, Split W, Rog-Malinowski M, Gredziak B. Spa treatment of shoulder pains at Swieradow. Neurol Neurochir Pol 1976;10(5):715-717. |
| Unable to source publication | Kulenkampff H-A, Reichelt A. Performance and effect of conservative treatments in case of tendinosis calcarea and supraspinatus syndrome. Orthopadische Praxis 1989;25(4):235-238. |
| Unable to source publication | Martinez Poza A, Hidalgo de Caviedes A, Blanco Pedraz JM. How to treat Volkmann's ischemic contraction. Rehabilitacion (Madr) 1977;11(3):303-308. |
| Unable to source publication | Matoba, T. The effects of physical therapy and balneotherapy on the patients with vibration disease. Journal of Japanese Association of Physical Medicine Balneology and Climatology 1978;42(1-2):39-41. |
| Unable to source publication | Mercanton G. Padey, A, Expert, JM, Liotard, J. L'experience de 3000 épaules en balnéotherapie: Un big-bang thermal. Kinésithérapie Scientifique 1997;(368):2-12. |
| Unable to source publication | Mihancea P, Mutiu G. Clinical and therapeutic aspects of radial nerve lesion. Archives of the Balkan Medical Union 2004;39(1):16-22. |
| Unable to source publication | Mucha C, Wannske M. Results of a controlled trial to physical therapy of the epicondylopathia humeri. Zeitschrift fur Physikalische Medizin Balneologie Med. Klimatologie 1989;18(3):137-147. |
| Unable to source publication | Nappi G, De Luca S, Genna A. Improvement of the general health status measures in patients submitted to thermal water and mud therapy for scapolo-humeral periarthritis. Medicina Clinica e Termale 2005;18(59):181-189. |
| Unable to source publication | Queneau P, Francon A, Graber-Duvernay B. Methodological reflections on 20 randomized clinical hydrotherapy trials in rheumatology. Therapie 2001;56(6):675-684. |
| Unable to source publication | Schmidt, KL. [Physical therapy and balneotherapy of arthritis].  Ther Umsch1991;48(1):46-51. |
| Unable to source publication | Scholtz, HG. [The hydrotherapy of painful stuff shoulder in the chronic stage]. Arch Phys Ther (Leipz) 1960;101-4. |
| Unable to source publication | Simon L, Blotman F. Exercise therapy and hydrotherapy. Clin in Rheum Dis 1981;7(2):337-347. |
| Unable to source publication | Simon L, Blotman F, Leroux JL, et al. [Rehabilitation and algodystrophies]. Rev Rhum Mal Osteoartic 1982;49(12):861-5. |
| Unable to source publication | Toomey R Grief-Schwartz R. Extent of whirlpool use in Canadian physiotherapy departments: a survey. Physiother Can 1986;38(5):277-278. |
| Unable to source publication | Vinel, P. [A thermal treatment of rheumatic diseases of the hands. The hand bath at Ax-les-Thermes]. Presse Therm Cli 1964;101:40-3. |
| **Wrong concept/intervention n=59** | |
| Wrong concept/intervention | Australian New Zealand Clinical Trials Registry [Internet]: Syndney (NSW): NHMRC Clinical Trials Centre, University of Sydney (Australia); 2005 - . Identifier ACTRN12619000045112. The Effect of Balneotherapy on Treatment of Subacute Suraspinatus Tendinitis; 2019 Jan 14 [cited 2023 April 05]; [about 8 pages]. Available from: <https://anzctr.org.au/Trial/Registration/TrialReview.aspx?id=376643&isReview=true> |
| Wrong concept/intervention | Beasley J, Ward L, Knipper-Fisher K, Hughes K, Lunsford D, Leiras C Conservative therapeutic interventions for osteoarthritic finger joints: a systematic review. J Hand Ther 2019;32(2):153. |
| Wrong concept/intervention | Becker TJ. Finger exercise device for adduction and opposition. Phys Ther 1976;55(6):615. |
| Wrong concept/intervention | Bender T. Balneotherapy: Efficacy and safety in osteoarthritis. Ann Rheum Dis 2016;75(Supp 2):40. |
| Wrong concept/intervention | Bender T, Bálint G, Prohászka Z, Géher P, Tefner IK. Evidence-based hydro- and balneotherapy in Hungary-a systematic review and meta-analysis. Int J Biometeorol 2014;58(3):311-323. |
| Wrong concept/intervention | Benini C, Rubino G, Paolazzi G, et al. Efficacy of mud plus bath therapy as compared to bath therapy in osteoarthritis of hands and knees: A pilot single-blinded randomized controlled trial. Reumatismo 2021;73(3):156-166. |
| Wrong concept/intervention | Cavanaugh JT. Effective upper extremity rehabilitation in the modern era. Tech Shoulder Elb Sur 2014;15(1):1. |
| Wrong concept/intervention | Chodakiewitz YG, Daniels AH, Kamal RN, Weiss AP. Post-traumatic Raynaud's phenomenon following volar plate injury. R I Medical J 2014;97(4):24-26. |
| Wrong concept/intervention | Chary-Valckenhaere I, Loeuille D, Kohler F. et al. Spa therapy in the treatment of chronic shoulder pain due to rotater cuff tendinopathy: rotatherm, a large randomized multicentre trial, Crenotherapie des douleurs chroniques de l'epaule par tendinopathies de la coiffe des rotateurs: rotatherm, un grand. Ann of Phys and Rehabil Med [Internet] 2012 [cited 2023 April 13]; 55(1):e352. Available from: <https://doi.org/10.1016/j.rehab.2012.07.893> |
| Wrong concept/intervention | Cioara F, Venter A, Birsan SD, et al. Rehabilitation after a distal radius fracture epiphyses in patients with osteoporosis. Osteoporosis International 2014;25(Supp 2):S415. |
| Wrong concept/intervention | ClinicalTrials.gov. [Internet] Bethesda (MD): National Library of Medicine (US). 2000 Feb 29 -. Identifier NCT02793401, Chronic Shoulder Pain Treatment: High Intensity Laser or Ultrasound; 2016 June 8 [cited 2023 April 05] [about 7 screens]. Available from: <https://clinicaltrials.gov/ct2/show/study/NCT02793401> |
| Wrong concept/intervention | Collins NJ, Hart HF, Mills KAG. Osteoarthritis year in review 2018: rehabilitation and outcomes. Osteoarthritis Cartilage 2019;27(3):378-391. |
| Wrong concept/intervention | Current Controlled Trials [Internet]. London: BioMed Central. [Date unknown] -. ISRCTN68246661, Supported fast track multi-trauma rehabilitation service; 2008 Aug 21 [Cited 2023 April 05]; [about 10 pages]. Available from <http://doi.org/10.1186/ISRCTN68246661> |
| Wrong concept/intervention | Devrimsel G, Turkyilmaz AK, Yildirim M Beyazal MS. The effects of whirlpool bath and neuromuscular electrical stimulation on complex regional pain syndrome. J Phys Ther Sci 2015;27(1):27-30. |
| Wrong concept/intervention | Dinçer U, Kaya E, Çakar E, Kıralp MZ, Dursun H. Effectiveness of comprehensive rehabilitation program and home-based exercise in middle and long term mastectomy related disability. Turk J Phys Med Rehabil 2007;53(4):138-143. |
| Wrong concept/intervention | Dionyssiotis Y, Dontas IA, Economopoulos D, Lyritis GP. Rehabilitation after falls and fractures. J Musculoskelet Neuronal Interact 2008;8(3):244-250. |
| Wrong concept/intervention | Forestier R, Francon A, Guillemin F. Spa therapy evaluation, an overview. Rhumatologie. 1997;49(4):141-150. |
| Wrong concept/intervention | Fortunati NA, Fioravanti A, Seri G, Cinelli S, Tenti S. May spa therapy be a valid opportunity to treat hand osteoarthritis? A review of clinical trials and mechanisms of action. Int J Biometeorol 2016;60(1):1-8. |
| Wrong concept/intervention | Gimigliano F, Iolascon G, Riccio I, Frizzi L, Gimigliano R. Post-surgical rehabilitative approach to fragility fractures. Aging Clin Exp Res 2013;25(1 Supp):S23-S25. |
| Wrong concept/intervention | Graber-Duvernay B, Forestier R, Francon A. Efficacy of the Berthollet technique at Aix les Bains spa on functional impairment in hand ostheoarthritis. A controlled therapeutic study. Rhumatologie 1997;49(4):151-156. |
| Wrong concept/intervention | Handoll HHG, Elliott J. Rehabilitation for distal radial fractures in adults. Cochrane Database Syst Rev 2015;9:CD003324. |
| Wrong concept/intervention | Handoll HH, Madhok R, Howe TE. Rehabilitation for distal radial fractures in adults. Cochrane Database Syst Rev 2002;(2):CD003324 |
| Wrong concept/intervention | Handoll HH, Madhok R, Howe TE. Rehabilitation for distal radial fractures in adults. Cochrane Database Syst Rev 2006;3:CD003324. |
| Wrong concept/intervention | Horoz L. Fluidotherapy in Patients With Distal Radius Fractures  NCT06272877, 2024 |
| Wrong concept/intervention | Horváth K, Kulisch Á, Németh A, Bender T. Evaluation of the effect of balneotherapy in patients with osteoarthritis of the hands: a randomized controlled single-blind follow-up study. Clin Rehabil 2012;26(5):431-441. |
| Wrong concept/intervention | Hui ACF, Wong S-M. Treatment options for carpal tunnel syndrome. Therapy 2005;2(3):455-463. |
| Wrong concept/intervention | Jerosch J, Wustner P. The effect of a sensorimotor exercise program in patients with subacromial pain syndrome. Unfallchirurg 2002;105(1):36-43. |
| Wrong concept/intervention | Kalichman LK, Vered E. [Results of an on-line survey of methods used by physical therapists for the management of hand osteoarthritis: pilot study]. Journal of the Israeli Physical Therapy Society. 2011;13(3):1. |
| Wrong concept/intervention | Karagulle M, Kardes S, Karagulle MZ. Real-life effectiveness of spa therapy in rheumatic and musculoskeletal diseases: a retrospective study of 819 patients. Int J Biometeorol. 2017;61(11):1945-1956. |
| Wrong concept/intervention | Kitchen, S. Faradic waterbaths in the treatment of hands. New Zealand Journal of Physiotherapy 1988;16(2):22-24. |
| Wrong concept/intervention | Koç C, Kurt EE, Koçak FA, Erdem HR, Konar NM. Does balneotherapy provide additive effects to physical therapy in patients with subacute supraspinatus tendinopathy? A randomized, controlled, single-blind study. Int J Biometeorol 2021;65;301–310. |
| Wrong concept/intervention | Kovács C, Pecze M, Tihanyi Á, Kovács L, Balogh S, Bender T. The effect of sulphurous water in patients with osteoarthritis of hand. Double-blind, randomized, controlled follow-up study. Clin Rheumatol 2012;31(10):1437-1442. |
| Wrong concept/intervention | Labie C, Runge N, Mairesse O, et al. Effectiveness of exercise - and physical activity interventions on fatigue in people with osteoarthritis: a systematic review with meta-analysis. Osteoarthritis Cartilage 2022;30(Supp 1):S33. |
| Wrong concept/intervention | Lanhers C, Pereira B, Gay C, Hérisson C, Levyckyj C, Dupeyron A, Coudeyre E. Evaluation of the efficacy of a short-course, personalized self-management and intensive spa therapy intervention as active prevention of musculoskeletal disorders of the upper extremities (Muska): a research protocol for a randomized controlled trial. BMC Musculoskelet Disord 2016;17(1):497. |
| Wrong concept/intervention | Marriott KA, Chopp-Hurley JN, Maly MR. A year in review: rehabilitation & outcomes. Osteoarthritis Cartilage 2019;27(Supp 1):S21. |
| Wrong concept/intervention | Mclaughlin P, Goddard N, Ahrens P, Subel B, Pollard D, Chowdary P. Managing at a distance - polytrauma in a patient with severe hemophilia A - a multidisciplinary case review. Haemophilia 2014;20(Supp 3):147. |
| Wrong concept/intervention | Metin Ökmen B, Ökmen K, Özkuk K, Uysal B, Sezer R, Koyuncu E. Comparison of the Efficacy of High Intensity Laser and Ultrasound Therapies in Chronic Shoulder Pain; Randomized Controlled Single Blind Study. Journal of Physical Medicine & Rehabilitation Sciences 2017;20(2):57-65. |
| Wrong concept/intervention | Morer C, Roques C-F, Francon A, Forestier R, Maraver F. The role of mineral elements and other chemical compounds used in balneology: data from double-blind randomized clinical trials. Int J of Biometeorol 2017;61(12):2159-2173. |
| Wrong concept/intervention | Nafai S, Stevens-Nafai E, Salman H. The Effectiveness of Hand Exercise and Thermal Modalities Agents in Managing Osteoarthritis and Rheumatoid Arthritis in Hands: Randomized Controlled Study. Am J Occup Ther 2017(Supp);71:241-241. |
| Wrong concept/intervention | Nguyen C, Lefevre-Colau M-M. Rehabilitation (exercise and strength training) and osteoarthritis: A critical narrative review. Ann Phys Rehabil Med 2016;59(3):190-195. |
| Wrong concept/intervention | Ozkuk K, Ates Z. Balneotherapy in the treatment of chronic shoulder pain: A randomized controlled clinical trial. Altern Ther Health Med 2020;26(1):18-24. |
| Wrong concept/intervention | Padey A. Rehabilitation after shoulder's arthroplasty - information for the patients. Revue du Rhumatisme Monographies 2010;77(3):253-263. |
| Wrong concept/intervention | Palazzo C, Revel M. Adhesive capsulitis. Simple to complicated medical treatments. Revue du Rhumatisme Monographies 2010;77(3):268-272. |
| Wrong concept/intervention | Pradhan S, Chiu S, Burton C, et al. Overall effects and moderators of rehabilitation in patients with wrist fracture: A systematic review. Phys Ther 2022;102(6):pzac032. |
| Wrong concept/intervention | Richter M. [Revision of failed resection arthroplasty of the CMC-1 joint using a costochondral graft]. Oper Orthop Traumatol 2021;33(3):216-227. |
| Wrong concept/intervention | Sayyid R, Uthman I. Hand osteoarthritis: An update on therapy. Arch Rheumatol 2015;30(2):150-158. |
| Wrong concept/intervention | Sen U, Karagulle M, Erkorkmaz U. The efficacy of balneotherapy in the patients with subacromial impingment syndrome. Turkiye Klinikleri Journal of Medical Sciences 2010;30(3):906-913. |
| Wrong concept/intervention | Sinclair M. Fingertips for the client. Hydrotherapy at home. Massage & Bodywork 2009, Nov/Dec:24(6):34-35. |
| Wrong concept/intervention | Sinclair M. Hydrotherapy in Your Practice. Massage & Bodywork 2020, Sep/Oct:70-77. |
| Wrong concept/intervention | Smart KM, Ferraro MC, Wand B, O’Connell NE. Physiotherapy for pain and disability in adults with complex regional pain syndrome (CRPS) types I and II. Cochrane Database Syst Rev 2022;(5). |
| Wrong concept/intervention | Swita M.; Szymonek P.; Talarek K.; Tomczyk-Warunek A.; Turzanska K.; Posturzynska A.; Winiarska-Mieczan A. Complex Regional Pain Syndrome after Distal Radius Fracture-Case Report and Mini Literature Review. Journal of Clinical Medicine / 2024;13(4):1122 |
| Wrong concept/intervention | Tander B, Cantürk F, Cengiz K, Durmuş D, Akyol Y. Are the physical therapeutic modalities really safe? Turk J Phys Med Rehabil 2005;51(4):131-133. |
| Wrong concept/intervention | Tefner IK, Kovács C, Gaál R, et al. The effect of balneotherapy on chronic shoulder pain. A randomized, controlled, single-blind follow-up trial. A pilot study. Clin Rheumatol 2015;34(6):1097-1108. |
| Wrong concept/intervention | Tenti S, Manica P. Cheleschi S, Fioravanti A. Sulfurous-arsenical-ferruginous balneotherapy for osteoarthritis of the hand: results from a retrospective observational study. Int J Biometeorol 2020;64:1561–1569. |
| Wrong concept/intervention | Toomey R, Grief-Schwartz R, Piper MC. Clinical evaluation of the effects of whirlpool on patients with Colles' fractures. Physiother Can 1986;38(5):280-284. |
| Wrong concept/intervention | Ucar D, Paker N, Bugdayci DS, Yalcinkaya EY. Comparison of the efficacy of whirlpool and paraffin treatments in women with symptomatic hand osteoarthritis. Turk J Phys Med Rehabil 2011;57(3):124-127. |
| Wrong concept/intervention | Wanczyk A, Pieniazek M, Pelczar-Pieniazek M. Method and results of rehabilitation for extensor tendon injury of fingers II-V in zone I and II. Ortopedia Traumatologia Rehabilitacja 2008;10(3):218-225. |
| Wrong concept/intervention | Yalcin G, Mulkoglu C, Gulmez S, Genc H. The effect of mirror therapy in the rehabilitation of flexor tendon injuries after primary surgical repair. Hand surgery & rehabilitation / 2024;43(1):101612 |
| Wrong concept/intervention | Zyluk A. Results of the treatment of posttraumatic reflex sympathetic dystrophy of the upper extremity with regional intravenous blocks of methylprednisolone and lidocaine. Acta Orthop Belg 2002;68(Supp):122-126. |
| **Wrong setting n=3** | |
| Wrong setting | Chary-Valckenaere I, Loeuille, D, Jay N, et al. Spa therapy together with supervised self-mobilisation improves pain, function and quality of life in patients with chronic shoulder pain: a single-blind randomised controlled trial. Int J Biometeorol 2018;62, 1003–1014. |
| Wrong setting | ClinicalTrials.gov. [Internet] Bethesda (MD): National Library of Medicine (US). 2000 Feb 29 -. Identifier NCT05332080, Telerehabilitation in Distal Radius Fracture; 2022 April 18 [cited 2023 April 05] [about 7 screens]. Available from: <https://www.clinicaltrials.gov/ct2/show/NCT05332080> |
| Wrong setting | Saul M, Battistella LR, Bazan M, et al. Guidelines: Breast cancer rehabilitation. PM R 2012;4(10 Supp 1):S211-S212. |
| **Wrong patient population n=24** | |
| Wrong patient population | Aquatic Therapy Association of Chartered Physiotherapists. Guidance On Good Practice In Aquatic Physiotherapy. Aquatic Therapy Association of Chartered Physiotherapists. Version 1.1, 2015. |
| Wrong patient population | Barker AL, Talevski JB, Morello, RT. Effectiveness of Aquatic Exercise for Musculoskeletal Conditions: A Meta-Analysis. Arch Phys Med Rehabil 2014;95(9):1776-1786. |
| Wrong patient population | Baumann FT, Reike A, Reimer V et al Effects of physical exercise on breast cancer-related secondary lymphedema: a systematic review. Breast Cancer Res Treat 2018; 170;1–13. |
| Wrong patient population | Bentley K, Jalil U, Bushi S. Intensive inpatient pain rehabilitation program for adolescents and young adults (age 18-21). PM R 2017;9(9 Supp 1):S140. |
| Wrong patient population | Castillo-Lozano R, Cuesta-Vargas A, Gabel CP. Analysis of arm elevation muscle activity through different movement planes and speeds during in-water and dry-land exercise. J Shoulder Elbow Surg 2014;23(2):159-165. |
| Wrong patient population | Effect of Exercise Intervention on Arthritis of Hand  CTRI/2024/03/063941, 2024 |
| Wrong patient population | De Peiza P. Optimizing shoulder function in cancer patients through the use of novel group-based pilot programming. Arch Phys Med Rehabil [Internet]. 2017[cited 2023 April 8];98(10):e88. Available from: <https://doi.org/10.1016/j.apmr.2017.08.278> |
| Wrong patient population | Dziedzic K, Jordan JL, Foster NE. Land-and water-based exercise therapies for musculoskeletal conditions. Clin Rheumatol 2008;22(3):407-418. |
| Wrong patient population | Falagas ME, Zarkadoulia E, Rafailidis PI. The therapeutic effect of balneotherapy: Evaluation of the evidence from randomised controlled trials. Int J of Clin Pract 2009;63(7):1068-1084. |
| Wrong patient population | Fujisawa H. Suenaga N, Minami A. Electromyographic study during isometric exercise of the shoulder in head-out water immersion. J Shoulder Elbow Surg 1998;7(5):491-494. |
| Wrong patient population | Geytenbeek, J. The evidence for aquatic physiotherapy for osteoarthritis. Aqualines: the news magazine/journal of the Hydrotherapy Association of Physiotherapists 2009;21(2):13-17. |
| Wrong patient population | Graca M, Alvarelhao J, Costa R, et al. Immediate effects of aquatic therapy on balance in older adults with upper limb dysfunction: An exploratory Study. Int J Environ Res Public Health 2020;17(24):9434. |
| Wrong patient population | Han S, Li T, Cao Y, Li Z, Mai Y, Fan T, Zeng M, Wen X, Han W, Lin L, Zhu L, Fu S, Bennell K, Hunter DJ, Ding C, Li L, Zhu Z. Quantitative analysis of effectiveness and associated factors of exercise on symptoms in osteoarthritis: a pharmacodynamic model-based meta-analysis. British journal of sports medicine / 2024;(0432520): |
| Wrong patient population | Heiller I. Physical therapy for neck and shoulder pain: Why, where and how often? Journal fur Mineralstoffwechsel 2007;14(4):137-142. |
| Wrong patient population | Kamioka H, Tsutani K, Mutoh Y, et al. A systematic review of nonrandomized controlled trials on the curative effects of aquatic exercise. Int J Gen Med. 2011;4:239-60. |
| Wrong patient population | Lauer J, Vigier S, Delage F, et al. Shoulder joint moment, work and power during slow underwater scapular plane abduction/adduction. Ann of Phys Rehabil Med [Internet]. 2016 [cited 2023 Apr 13];59(Supp):e118. Available from: <https://doi.org/10.1016/j.rehab.2016.07.266> |
| Wrong patient population | Lauer J, Vilas-Boas JP. Rouard AH. Shoulder mechanical demands of slow underwater exercises in the scapular plane. Clinical Biomechanics 2018;53:117-123. |
| Wrong patient population | Manara M, Bortoluzzi A, Favero M, et al. Italian society for Rheumatology recommendations for the management of hand osteoarthritis. Reumatismo 2013;65(4):167-185. |
| Wrong patient population | Moore-Higgs G. Aquatic rehabilitation for shoulder dysfunction after breast cancer-a single subject design. Oncol Nurs Forum 2009;36(3):57-58. |
| Wrong patient population | Odynets T, Briskin Y, Yefremova A, Goncharenko I. The effectiveness of two individualized physical interventions on the upper limb condition after radical mastectomy. Physiotherapy Quarterly 2019;27(1):12-17. |
| Wrong patient population | Schencking M, Wilm S, Redaelli M. A comparison of Kneipp hydrotherapy with conventional physiotherapy in the treatment of osteoarthritis: A pilot trial. J Integr Med 2013;11(1):17-25. |
| Wrong patient population | Suomi R, Collier D. Effects of arthritis exercise programs on functional fitness and perceived activities of daily living measures in older adults with arthritis. Arch of Physi Med and Rehabil 2003;84(11):1589-1594. |
| Wrong patient population | Suomi R; Lindauer S. Effectiveness of arthritis foundation aquatic program on strength and range of motion in women with arthritis. J Aging Physical Acct 1997;5(4):341-351. |
| Wrong patient population | Welsh Physiotherapy Advisory Group. All Wales Evidence Based Guidance for Access to Hydrotherapy for NHS Patients in Wales. Welsh Physiotherapy Advisory Group. WPhLAG Vers 3, 2016. |
