# Supplementary figures and images for: Aquatic exercise interventions in the treatment of musculoskeletal upper extremity disorders: A scoping review
Source: Clin Rehabil. 2025 Feb 2;39(5):565–79. doi: 10.1177/02692155251315078 (PMC12099020; doi:10.1177/02692155251315078)

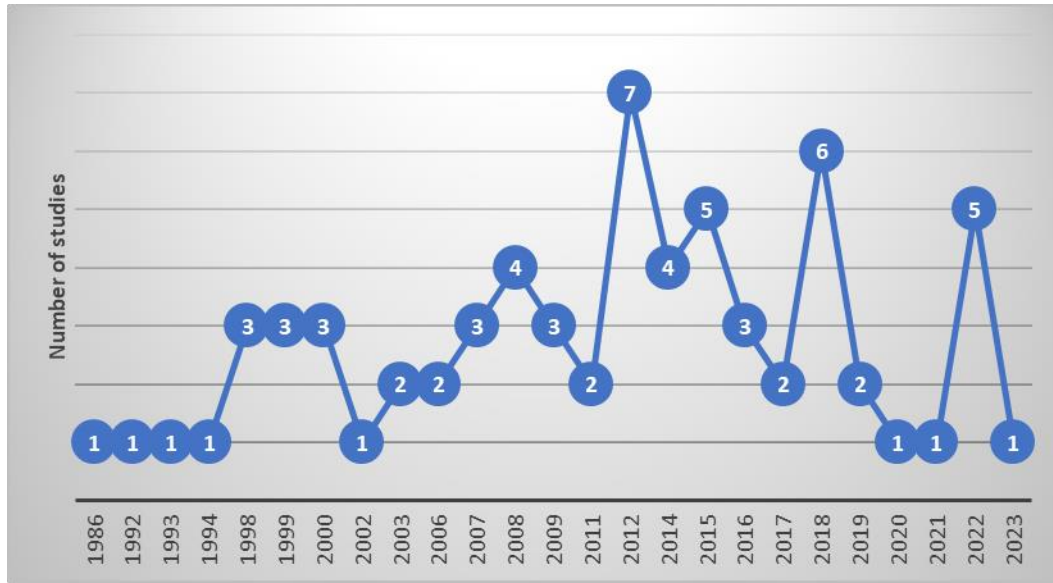

Supplement: sj-pdf-3-cre-10.1177_02692155251315078 - Supplemental material for Aquatic exercise interventions in the treatment of musculoskeletal upper extremity disorders: A scoping review [file sj-pdf-3-cre-10.1177_02692155251315078.pdf]

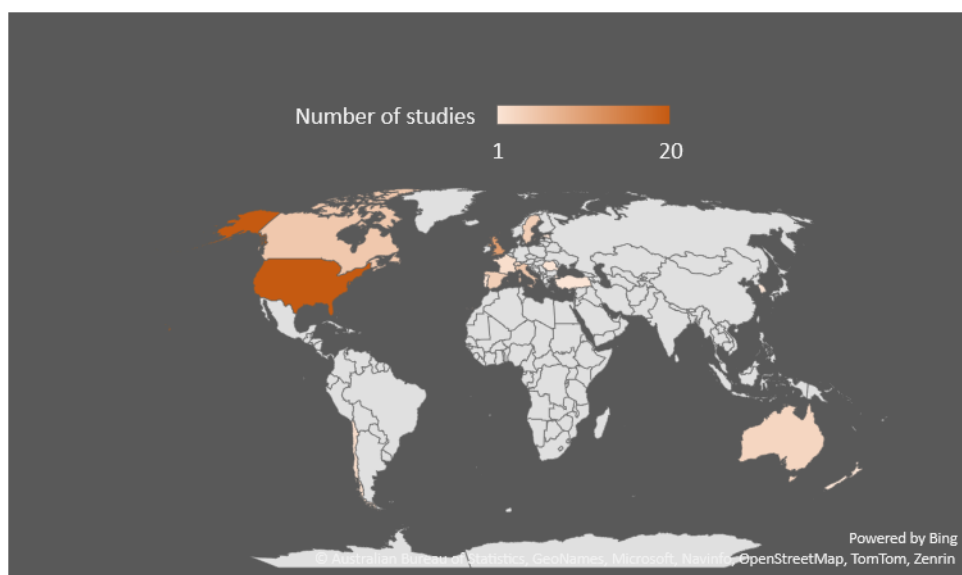

Supplement: sj-pdf-4-cre-10.1177_02692155251315078 - Supplemental material for Aquatic exercise interventions in the treatment of musculoskeletal upper extremity disorders: A scoping review [file sj-pdf-4-cre-10.1177_02692155251315078.pdf]

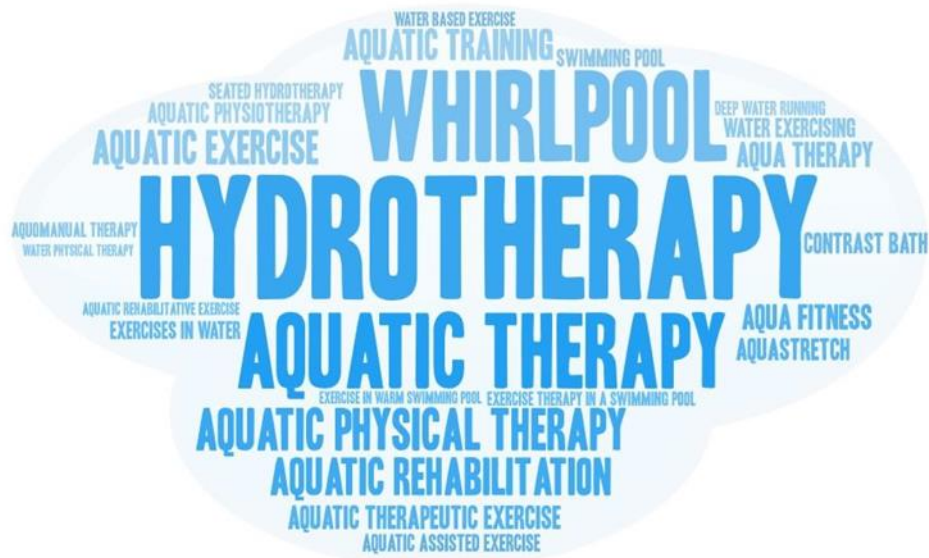

Supplement: sj-pdf-6-cre-10.1177_02692155251315078 - Supplemental material for Aquatic exercise interventions in the treatment of musculoskeletal upper extremity disorders: A scoping review [file sj-pdf-6-cre-10.1177_02692155251315078.pdf]
