# Supplementary material for: Aquatic exercise interventions in the treatment of musculoskeletal upper extremity disorders: A scoping review
Source: Clin Rehabil. 2025 Feb 2;39(5):565–79. doi: 10.1177/02692155251315078 (PMC12099020; doi:10.1177/02692155251315078)
Supplement: sj-docx-5-cre-10.1177_02692155251315078 - Supplemental material for Aquatic exercise interventions in the treatment of musculoskeletal upper extremity disorders: A scoping review [file sj-docx-5-cre-10.1177_02692155251315078.docx]

**Characteristics of included studies**

| Author, Year, Country | | Aim of study/purpose | Study design | Total number of participants | Gender | | Age | | Upper extremity condition | | Ethnicity | | Comorbidities | | Time since injury/Chronicity | | | Author conclusions | | |
| --- | --- | --- | --- | --- | --- | --- | --- | --- | --- | --- | --- | --- | --- | --- | --- | --- | --- | --- | --- | --- |
| Doig 2008, Australia | | The purpose of this systematic review was to identify and summarise the evidence from randomised controlled trials supporting the use of hydrotherapy (aquatic exercise, pool therapy) in acute and chronic medical conditions. | Systematic review | N/A | N/A | | N/A | | Fracture of proximal humerus | | N/A | | N/A | | N/A | | | NA | | |
| Delbrouck 2003, France | | Identify criteria for referral to day hospitalization or in-patient after shoulder rotator cuff surgery.  To compare 2 types of hospitalization to know if the offer the same guarantees of results, in terms of pain and function while avoiding complications. | Cohort study | 76 shoulders included for 71 patients 53 - boarding  23 day hospital | 41m, 30f - in-pt: 25m, 24f; day hosp: 16m, 6f | | Av age 53.2 +/- 7.6 yrs, in-pt: 52.7+/-8 yrs; day hosp: 55+/-5 yrs | | Rotator cuff repair | | N/R | | N/R | | As soon as skin had healed and manually dry. | | | Comparisons between patients did not show any significant difference in terms of range of motion and complications | | |
| Woolfenden 1994, USA | | Describes how aquatic physical therapy can be used to treat five common orthopaedic conditions of the extremities. | Text and opinion | N/A | N/A | | N/A | | Shoulder injury Lateral epiondylitis | | N/A | | N/A | | N/R | | | The aquatic environment is an excellent medium in which to rehabilitate orthopaedic patients. Exercises can be easily progressed from assisted to highly resisted. To take complete advantage of water, effective treatment involves working the whole body, not just the injured area | | |
| Castelhano 2022, UK | | This study compares two pathways for patients undergoing MUA for FS: one in a NHS setting where physiotherapy advice only is given to the patient, and the other in an independent hospital setting where supervised hydrotherapy and physiotherapy occur postoperatively. | Non-randomised experimental study | 248, 129 MUA, 119 MUA+physio (Only 119 had aquatic intervention) | MUA: 58m, 71f; MUA+Physio: 59m, 60f (aquatic population) | | Aged 16-80 years | | Frozen shoulder (primary or secondary) | | N/R | | Diabetes disease status, primary versus secondary procedure | | N/R | | | Following MUA for FS, a statistically significant increase in OSS was detected in patients receiving post procedure physiotherapy compared with advice alone. There was no difference in recurrence rates. The increase in OSS (3.2) is below the minimal clinically important difference, raising questions regarding the relative importance of post procedure physiotherapy in a resource-limited environment. | | |
| Liotard 1999 | | To report experience of rehabilitation post shoulder arthroplasty | Case series | 130 | N/R | | Average ages between clinical subgroups 57-73 | | Shoulder arthroplasty for:  osteoarthritis 58 cases, rheumatoid arthritis 21, old fractures 16, 34 other causes: osteonecrosis (5cases), revision surgery (12cases), locked posterior dislocations (4 cases), cuff tear arthropathies (5 cases), complexes fractures (8 cases), arthritis after radiotherapy (1case), necrosis after large cuff tear (1case). | | NR | | NR | | End of second post-operative week and after clip or stitches have been removed | | | This development using the pool must be run in parallel with two ideas:(a)the results of rehabilitation should not, strictly speaking, be judged until after the first 3 months and not the first 3 weeks; and (b)an assessment of the cost, including all the different means of administering post-operative rehabilitation, is difficult, but it  is clear that as the years have gone by, the Centre provides a short extension to the post-operative period after a standard arthroplasty but with a functional result when the patient leaves. | | |
| Wilk 2000, USA | | We will discuss the postoperative rehabilitation programs followed after various rotator cuff repair surgeries. | Text and opinion | N/A | N/A | | N/A | | Rotator cuff repair | | N/A | | N/A | | All sizes of tears - 3-4 weeks | | | In this article, we have described three different rehabilitation programs based on several key factors, and have emphasized immediate motion, early dynamic glenohumeral joint stability, and a gradual restoration of rotator cuff muscular strength.  (i.e. nothing directly related to hydrotherapy) | | |
| Klintberg 2009, Sweden | | To evaluate the out-come of two different physiotherapy treatment protocols for patients with full-thickness rotator cuff repair up to two years after surgery. | Prospective, Randomised pilot study | 14 (n=7 progressive rehab; n=7 traditional group) | 9m, 5f | | Median age 55 (40-64) | | Rotator cuff repair with subacromial decompression  (full thickness tear) | | N/R | | N/R | | Progressive group - start at 4 weeks post op Aquatic training programme 1 & at week 10 post op start Aquatic training programme 2.  Traditional group - 10 weeks post op Aquatic training programme 1 & week 16 post op for Aquatic training programme 2. | | | A more proactive an progressive physiotherapy programme is associated with slightly faster recovery time.  The present study showed that the progressive protocol produced no adverse effects compared with the traditional protocol. We feel that it is important to find evidence of how rehabilitation can be made more effective. | | |
| Zanazzo 2014, Italy | | The aim of this study was to compare the effectiveness of a traditional and an aquatic post-surgery rehabilitation program of the rotator cuff. | Abstract of a RCT | 20 (Aquatic =10), (traditional = 10) | N/R | | both groups 60+5y. | | Rotator cuff repair | | NR | | NR | | 15 days | | | Although the water-based program did not result more effective than the traditional program at the end of the three months, the aquatic rehabilitation seemed to improve shoulder ROM faster than the traditional one and to be better tolerated by the patients. Itâ€™s authorsâ€™ opinion that both evidences may increase the adherence to the program during the rest of the rehabilitation program | | |
| Grigware 2015, USA | | The purpose of this case report was to report upon the outcomes of aquatic therapy on the biomechanical and functional retraining of a patient following a rTSA. | Case report | 1 | 1f | | 68 | | Reverse total shoulder arthroplasty (rTSA) | | N/R | | N/R | | 8 weeks | | | This patient improved mechanically and functionally during her episode of care and those improvements can be seen in her increased active range motion, increased strength, decreased pain, decreased activity limitations, and decreased participation restrictions. Future studies on aquatic therapy in joint replacements, particularly procedures involving the upper extremity, would be beneficial to the evidence-based treatment of our actively aging, longer-living population. | | |
| Bufete 2015, USA | | Guidance on treating post-surgical shoulder patients in the pool | Text and opinion | N/A | N/A | | N/A | | post-surgical shoulder (such as arthroscopic debridement (cleaning out), labral repair, rotator cuff repair, and shoulder replacement) | | N/A | | N/A | | NA | | | With these principles, your patient will progress to a point where he/she can regain strength and return to full function. | | |
| Appunni 2012, Canada | | The purpose was to inform about postoperative Physical therapy following rotator cuff repair, with the following objectives: To systematically review the content of clinical research, which addresses various physical therapy programs. To describe validity and responsiveness of different testing positions for goniometric measurement of shoulder active external rotation. To pilot test study procedures and estimating effects of a land-based and an aquatic exercise program. | PhD 2 areas of PhD relevant to topic- Systematic review & Prospective randomized pilot study | 12 | land plus aquatic - 3m, 3f;  land - 4m, 2f | | mean age: 49 (land plus aquatic 51 (land) | | Rotator cuff repair - mean tendon size tear 1.4cm | | N/R | | N/R | | Start of post operative 3rd week | | | Systematic review: Our current review, three studies compared aquatic exercises with land-based exercises, and two studies agreed that aquatic exercises were a useful adjunct to rehabilitation There is weak overall evidence to define the use of physical therapy interventions, and small to moderate evidence to support progressive exercise programs as a core element of rehabilitation with the potential addition of adjunctive components including continuous passive movements, aquatic exercises, and neuromuscular electrical stimulation.   Prospective randomized pilot study:  This current study suggests that the combination of land-based exercise and aquatic exercise as an adjunct may be equally effective in improving shoulder AROM and HRQOL, when compared to land-based exercises only.  Areas to be examined include clinician, researcher, and patient participation; and appropriate incentives that would fit within acceptable ethical behavior. Answering these questions may help conduct a future randomized study, to find statistical significance between land-based exercise program and potential adjunctive aquatic exercise program for patients following rotator cuff repair. | | |
| Leininger 2018, USA | | To determine the effectiveness of aquatic therapy (AT) on increasing range of motion (ROM) and decreasing pain in adults with shoulder pathologies. | Systematic review | NA | N/A | | N/A | | Shoulder pathologies | | N/A | | N/A | | N/A | | | There is moderate to strong preliminary evidence suggesting use of AT as an adjunct to increase ROM and decrease pain in patients with SIS and s/p RCR. In addition, AT was found to improve both sleep quality and over-all function. Limitations include small sample sizes, varied outcome measures, and lack of larger RCTs. Future research is necessary to determine the optimal protocol in the use of AT to increase shoulder ROM and decrease pain. | | |
| Huss 2014, USA | | N/R | Case report | 1 | 1m | | 49 yrs | | Frozen shoulder | | N/R | | Type 1 DM | | 11 months - traditional aquatic therapy  20 months - aquastretch | | | Further research needed to validate aquastretch frozen shoulder treatment efficacy with a larger patient population.  Patient did not experience 'treatment induced' discomfort compared to traditional manual mobilizations. | | |
| GraÃ§a 2019, Portugal | | Chapter II - systematic review  Chapter intends to systematically review and discuss the different approaches that have been used, combining aquatic and dry land interventions for rotator cuff injury and draw conclusions based on these results. | Systematic review | N/A | N/A | | N/A | | Rotator cuff injury | | N/A | | N/A | | N/A | | | No strong conclusion can be made due to the high heterogeneity in rehabilitation protocols, times of assessment, included samples and outcome measurements used within the available studies. Further research on rotator cuff injury rehabilitation is needed combining programs for patients with higher level of pain and restriction of range of motion, supported by the outcomes on patientsâ€™ beliefs and responses to clinical changes. Additionally, further investigation on aquatic exercises is required to better develop aquatic therapy protocols for implementation in clinical practice. | | |
| Yeomans 2012, UK | | to employ a validated outcome measure to assess the outcomes of aquatic therapy treatment sessions for various musculoskeletal conditions and pathologies. | Audit | total = 23, shoulder = 2 | NR | | NR | | Shoulder | | N/R | | N/R | | N/R | | | Even though the data collected will only qualify as the smallest of pilot sized studies, it suggests that aquatic therapy delivers improvements in patient defined goals in terms of symptom improvement and a return to function. This is excellent news in maintaining support for the continued use and promotion of aquatic therapy as a clinically effective form of physiotherapy intervention. | | |
| Turnbull 2016, UK | | N/R | Case report | 1 | 1f | | 48 | | Surgery - extensive debridement of her right elbow, arm and axillary region, skin grafts & surgical releases of the forearm and elbow. | | N/R | | Smoker | | N/R | | | She returned to work and had achieved her goals. She also avoided surgical release of biceps. Avoidance of surgical interventions is important, for patients in terms of avoiding discomfort and inconvenience and for commissioners in terms of saving costs. | | |
| Smith 2015, Australia | | The aim of this review was to evaluate the published evidence regarding the use of Aquatic Therapy to assist cancer survivors to rehabilitate and manage adverse out-comes following treatment. Secondary aims were to identify considerations for exercise in an aquatic environment. | Literature review | N/A | N/A | | N/A | | Breast cancer | | N/A | | N/A | | N.A | | | This review has highlighted that there is limited research currently available evaluating Aquatic Physiotherapy and its application to Cancer Rehabilitation. The initial research findings indicate that the aquatic environment may offer clinicians and survivors a safe and well tolerated environment in which to improve many of the symptoms which cancer survivors experience. These include lymphedema, cancer-related fatigue, neck and shoulder pain, reduced upper limb strength and limited movement. | | |
| Maynard 2000, UK | | NR | Case report | 2 - only 1 relevant | 2f in total  Case study 1 - 1f | | Case study 1 - 87 | | Impacted fractured neck of humerus | | NR | | NR | | 4 months | | | These treatment programmes are by no means exhaustive, and are in no way meant to be prescriptive. Hopefully they will serve to stimulate ideas and discussion in the many ways water can be used to affect this fascinating ares of the body. | | |
| Hall 2006, UK | | NR | text and opinion | NR | NR | | NR | | CRPS | | NR | | NR | | NR | | | In practice, hydrotherapy for CRPS is used to ease movement, relieve pain, aid relaxation and act as a desensitisation device aiming to normalise sensory input.  Despite the significant barriers CRPS patients face to exercising in hydrotherapy pools. it os perceived as a vital element in the battle to regain health. | | |
| Carter 2008 | | N/R | Text and opinion | N/A | N/A | | N/A | | fractures - with or without open reduction and internal fixation subluxations / dislocations - includng post manipulation under anaesthetic arthroscopic sub acromial decompression / acromioplasty arthroplasty - hemi/total rotator cuff repairs stabilisations e.g. SLAP lesion repair of Putti-Platt repair nerve pasly | | N/A | | N/A | | N/R | | | Therefore. aquatic physiotherapy is a means towards early active motion for people post-surgery or for people with shoulder injuries. | | |
| RBR-59nbtf 2020, Chile | | To assess the wrist functionality prior to treatment and after treatment. | Randomised controlled trial | 37 | N/R | | 60+ | | Distal radius fracture | | N/A | | N/A | | N/R | | | N/A | | |
| Fisken 2015, New Zealand | | The purpose of this randomized controlled trial was to investigate the effects of an aqua fitness programâ€”compared with a seated, hydrotherapy type programâ€”on strength, function, balance, fear of falling, and perceived quality of life among older adults with OA | Randomised controlled trial | 17 with hand OA Aqua fitness 9  Control group 8 | AF 18f/1m: HT 15f/1m | | AF 71.9yrs (7.3); HT 70.4 (6.5) | | Hands/finger OA | | AF 15 NZ European, 1 Maori, 3 other; HT 13 NZ European, 3 Other Reported for whole group - not just those with hand OA | | N/R | | duration OA symptoms: AF 13.1yrs (9.3): control 17.6 yrs (10.9); joints affected by OA: AF hands/fingers 9; control hands/fingers 8; | | | Further investigation into the effects of aqua-based exercise on handgrip strength among older adults with hand OA is warranted.  Aqua fitness may offer a number if positive benefits for older adults with OA | | |
| Schrepfer 1998, USA | | The purpose of this review is to describe correct therapist and patient positioning during performance of glenohumeral joint mobilization, manual stretching of the shoulder and manual resistive exercise of the shoulder in water. | Text and opinion | N/A | N/A | | N/A | | N/R | | N/A | | N/A | | N/R | | | Aquatic manual techniques for the shoulder represent a valuable adjunct to more traditional land-based treatment. | | |
| Watts 2007, USA | | To identify evidence that exists to support the treatment of orthopaedic UE injuries with aquatic therapy | Literature review | N/A | N/A | | N/A | | Orthopedic upper extremity injury | | NA | | N/A | | N/A | | | Early activation of ROM, joint mobility, muscle strength, proprioception and core strength, reduced pain, reduced risk of re-injury and use of equipment to increase resistance were all factors supporting aquatic physical therapy intervention for UE injuries. | | |
| Prins 1999, USA | | The purpose of this article is to discuss the manner in which aquatic physical therapy is used for the treatment of common athletic injuries | Text and opinion | NA | N/A | | N/A | | Shoulder:  Fractures Subacromial bursitis, inflammation tendons Calcifying tendinitis  Rotator cuff tears Subluxations and dislocations  Elbow and RU joint  Contusions Dislocations Fractures Soft tissue damage   Wrist & hand injuries | | N/A | | N/A | | N/R | | | Aquatic therapy provides a unique environment for promoting normal movement patterns and building strength early in the course of treatment. These changes usually are accomplished in the water, where the risk of further injury is reduced. This frequently is accompanied by a reduction in pain and perceived discomfort. Aquatic therapy in many cases is the only option for rehabilitation when land-based programs have not provided satisfactory results. | | |
| Clark 2011, USA | | NR | Case report | 1 | 1m | | 19 | | Bristow-Latarjet procedure for recurrent shoulder dislocations | | N/R | | N/R | | N/R | | | There are many ways to assist a patientâ€™s recovery with aquatic exercise, but an underlying factor that needs to be considered is whether their specific sport is based on land or in water. If an athleteâ€™s sport is land-based, they will need to eventually train on land to effectively return to sport. | | |
| Wagner 2012 | | To perform a search of literature to identify evidence that exists to support the effective use of aquatic physiotherapy as a treatment technique for rotator cuff injuries. To evaluate whether aquatic physio-therapy helps reduce pain for people with rotator cuff injuries and to consider whether aquatic physiotherapy induces a faster recovery of range of motion after rotator cuff injuries | Literature review | N/A | N/A | | N/A | | Rotator cuff injuries | | N/A | | N/A | | N/A | | | The findings suggest that there is some evidence to support the use of aquatic physiotherapy for treatment of rotator cuff injuries however this conclusion has been based on studies of lower scientific merit. There is a definite need for further study within the area of aquatic physiotherapy and shoulder rehabilitation for rotator cuff injuries.  Aquatic physiotherapy can be considered effective as a form of exercise. It seems to reduce pain, and improve ROM more quickly following rotator cuff injuries. | | |
| Palmer 1998, USA | | To demonstrate the ability to restore the functional mobility and strength of a shoulder with a full thickness supraspinatus tear by the application of aquatic therapeutic exercises | Case report | 1 | 1f | | 78 | | Full thickness supraspinatus tear | | Caucasian | | Bilateral carpal tunnel release  Degenerative disc disease of the lumbar spine, Dercumâ€™s disease, sacral laminectomy S1-2 with excision of neurofibroma, OA lumbar spine, spinal stenosis Bilateral knee OA | | 6.5 months | | | demonstrates successful 3 months application of aquatic therapeutic exercises for non-surgical management of a full thickness rotator cuff tear | | |
| Thein 2000, USA | | To describe the application of aquatic rehabilitative exercise to injuries of the upper extremity. | Text and opinion | N/A | N/A | | N/A | | Athletes with: Elbow or glenohumeral dislocation Post surgery  Tendinitis or impingement. Injuries Pain for any reason Shoulder instability | | N/A | | N/A | | Acute or chronic | | | The pool is an effective medium for many aspects of the elite athlete's training and rehabilitation program. An understanding of the differences between land-based and water-based exercise allows the clinician to establish a comprehensive and effective rehabilitation program for the athlete. | | |
| Ahmed 2018, Canada | | To identify evidence in the literature on the therapeutic benefit of aerobic exercise on myofascial pain. | Systematic review | N/A | N/A | | N/A | | Myofascial pain | | N/A | | N/A | | N/A | | | N/A | | |
| Gutierrez-Espinoza 2019, Chile | | To describe the short term effect of a physical therapy program in function improvement and pain reduction in patients older than 60 years with complex regional pain syndrome (CRPS) type I after distal radius fracture (DRF) treated conservatively. | Case series | 54 | 8m 46f | | 65.3 ±3.9 | | CRPS after DRF | | N/R | | N/R | | 5.5Â±1.2 weeks | | | A physical therapy program based on hydrotherapy, manual therapy, and exercises in a short term improves the function and reduces the pain in patients older than 60 years with CRPS I after DRF treated conservatively. | | |
| Gliga 2022, Romania | | The aims of this study were to (1) investigate the effects of personalized physiotherapy on shoulder JPS among individuals with post-traumatic injury to the joint and (2) investigate whether additional aquatic-based therapy would have significant effects on shoulder JPS compared with only land-based rehabilitation. | Randomised controlled trial | 44 total  22 received hydrotherapy | Control:9F; 13M Experimental (hydrotherapy): 6F; 16M | | 48.05+/-9.02 (experimental) 44.50+/- 10.11 (control) | | 12 proximal humeral fracture 7 distal clavicle fractures  3 ACJ dislocations | | N/R | | N/R | | Less than 5 months | | | The findings of this study indicate that shoulder JPS can be significantly improved after only four weeks of personalized physiotherapy among individuals with post-traumatic injury to the joint. However, the lack of significant benefits from additional aquatic-based therapy indicates the lack of value for shoulder JPS for such exercises. Further research is required to assess whether aquatic-based therapy can provide benefits overland-based therapy on other relevant outcomes such as muscular strength and the joint range of motion | | |
| Dufournet 2022, Switzerland | | The purpose of this study was to compare the clinical and radiological outcomes of arthroscopic RCR over a period of two years between two different post-operative rehabilitation modalities: aquatic therapy and land-based therapy. | Randomised prospective study | 86 patients in total Aquatic therapy (n= 44) or standard rehabilitation (n= 42) | Aquatic therapy: 61.9% male 38.1% 27m and 17f  Standard therapy:  25m 17f | | Aquatic Therapy (55.0+-10.4) Standard therapy (58.5+-9.8), | | Small to large supraspinatus and or infraspinatus rotator cuff repair | | N/R | | N/R | | 10 days | | | When initiated immediately after surgery, aquatic therapy did not yield superior clinical or functional outcomes compared to land-based rehabilitation after arthroscopic RCR. Further studies are required to better define when the aquatic therapy should begin and on which patients in order to optimize both patient outcomes and unnecessary costs. | | |
| Hoyrup 1986, Canada | | The purpose of this study was to establish whether whirlpool baths can be used safely in the treatment of hand injuries.  The study was designed to determine the effects of whirlpool treatments during which the hand is dependent, as compared to wax treatment during which the hand is elevated, and to determine the effects of active exercise during the treatment. | Randomised controlled trial | 42 - across 4 groups 10 of which were in whirlpool & exercise group | 10m in whirlpool and exercise group  All participants were male | | Aged between 17-66 (mean=38, SD=15) (over all groups) | | Traumatic hand injury | | N/R | | N/R | | N/R | | | No adverse effects on oedema using whirlpool Significant improvements in ROM and pain using both wax or whirlpool. | | |
| Revay 1992, Sweden | | The aim of this study was to elucidate if swimming pool training added to instruction on self-training gave better results than instruction on self-training alone. | Randomised controlled trial | 48 in total  25 in swimming pool group | 39f, 9m - total  20f 5m swimming pool group | | Mean age 62 | | 2 fragment fracture of humerus at level of the surgical neck; or with 3-4 fragment fractures (non-displaced) or with less than 1cm displacement or more than 45 degrees angulation | | NR | | NR | | 5-10 days post fracture | | | Instruction for self-training with appropriate control procedures is an efficient way of treating patients. Added training in the swimming pool did not give better results. | | |
| Speer 1993, USA | | N/R | Text and opinion | N/A | N/A | | N/A | | Shoulder injury or surgery  Mentions adhesive capsulitis and rotator cuff repair | | N/A | | N/A | | NA | | | The maintenance of a patientâ€™s confidence in the ability to actively use the shoulder in varied dysfunctional states early in the course of postoperative rehabilitation seems to be a central tenet for successful total rehabilitation of the shoulder. This factor can be greatly enhanced by performing motion exercises in water, which creates a safe environment for rehabilitation of the shoulder. Water provides bouyancy to the arm, which can ease the accomplishment of the active tasks and may provide a component of enhanced proprioceptive feedback that may, in turn, aid the rehabilitative progression. | | |
| Caniggia 1998, Italy | | N/R | Text and opinion | N/A | N/A | | N/A | | Shoulder arthroplasty | | N/A | | N/A | | Recommends hydrotherapy prior to arthroplasty and from 2 weeks post arthroplasty. | | | ....the quality of rehabilitation following shoulder arthroplasty has greatly improved:undoubtedly the introduction of physical therapy in water has shortened the time necessary to recover range of motion and girdle function. | | |
| Binkley 2002, USA | | Presents exercise regimens that can be incorporated into current orthopedic rehabilitation or reconditioning programs for upper extremity athletes | Text and opinion | N/A | N/A | | N/A | | Shoulder injury / surgery - not specified | | N/A | | N/A | | Acute injury / post surgical onwards but no specified timescale | | | Using water in shoulder rehabilitation increases the effectiveness and transition to full participation in sport. Using water as resistance improves muscle strength and endurance by increasing the force of the movements and the ability to perform repetitive movements. | | |
| Liotard 2003, France | | This article outlines our hydrotherapy based rehabilitation regime. | Text and opinion | N/A | N/A | | N/A | | Rotator cuff repair or shoulder arthroplasty | | NA | | NA | | Day 4 postoperatively | | | Hydrotherapy following rotator cuff repair and shoulder arthroplasty is a successful method of regaining shoulder mobility and function. This method minimizes pain induced by postoperative rehabilitative efforts and does not require any additional strengthening exercises. Furthermore, the warm-water pool employed stimulates proprioceptive feedback while minimizing nociceptive function. The clinical success of this method, which has been implemented in over 3500 patients, attests to its role in postoperative rehabilitation of the shoulder. | | |
| Sokk 2007, Estonia | | Investigate the effect of 4 week individualized rehabilitation in combination of exercise therapy in swimming pool and gymnasium, massage and electrical therapy on shoulder muscle strength and fatiguability in patients with frozen shoulder syndrome. | Case control study | 20 total  10 with frozen shoulder | Total: 14f, 6m  FS group: 7f 3m | | FSS Grp: mean 50.2 +/-4.6; Control: 49.8 +/-4.6 years; 34-68 years range | | Frozen shoulder syndrome | | N/R | | NR | | 3-6 months | | | It was concluded that in patients with FSS, 4-week rehabilitation decreased shoulder pain and improved MVC force of the shoulder flexors and isometric working capacity of these muscles during endurance test. | | |
| Labriola 2008, USA | | The following article details our experience with reverse total shoulder arthroplasty in the senior athlete,  including patient presentation, preoperative imaging, surgical technique, sport-specific shoulder rehabilitation, and short-term outcomes. | Text and opinion | N/A | NA | | NR | | Reverse total shoulder arthroplasty with a range of shoulder conditions | | N/A | | N/A | | 3-4 weeks post operatively | | | After sport-specific rehabilitation programs, non contact senior athletes are able to return to their sport without limitations by approximately 6months postoperatively. Short-term outcomes are favorable, with most senior athletes returning to play with no current evidence of increased risk of component dislocation, loosening, or failure. Long-term outcome studies are needed to further define the results of reverse total shoulder arthroplasty in this population. | | |
| Brady 2008, Australia | | To investigate the feasibility of a combined aquatic and land-based physiotherapy programme in the post-operative rehabilitation of RC tears | Feasibility study | 18 | 11 male, 7 female; Aquatic grp 67% male; Land group 50% male | | mean 55 years (range 26-79, SD 11.42 yrs); Aquatic grp: 56.3 SD9.06 (41-67); land grp: 53.5 SD16.02 (26-69) | | Rotator cuff repair (small, medium, large and massive) | | N/R | | N/R | | Between 3-12 months | | | The implementation of a combined aquatic and land-based physiotherapy programme following surgical repair of the rotator cuff is feasible and presents a potential viable alternative to conventional land-based exercise with comparable outcomes | | |
| Ainsworth 2007, UK | | To synthesise the available research literature on the effectiveness of exercise therapy for full thickness tears of the rotator cuff. | Systematic review | N/A | N/A | | N/A | | full thickness rotator cuff tendor tear | | N/A | | N/A | | N/A | | | N/A | | |
| Janssen 2009, USA | | To determine the effects of specific contrast bath protocols on hand volume in patients diagnosed with Carpal Tunnel Syndrome | Randomised controlled trial | Pre-op: 58 (contrast bath + ex 18, contrast bath only 22, ex only 18) Post-op: 56 (CB+E 19, CB 23, Ex 14)  In total 37 had contract bath & exercise (18 pre-surgery & 19 post surgery) | 36 (62%)f, 22 (38%m) | | Average 51.5 years, range 31-82. | | Carpal tunnel syndrome or open surgical decompression of the carpal tunnel syndrome | | N/R | | N/R | | N/R | | | Contrast baths as described are not clinically effective in changing hand volume in pre- and postoperative CarpalTunnel Release patients. | | |
| Conti 2009, Italy | | N/R | Text and opinion | N/A | N/A | | N/A | | Rotator cuff repair | | N/A | | N/A | | Once sutures removed | | | Evident from the analysis set out above is the consensus about how post-operative rehabilitation constitutes a progressive, integrated and personalised process (rather than a â€œprotocolâ€) in which a fundamental role is played by the passage of information between the surgeon and the physiotherapist, as well as the sharing of knowledge regarding the characteristics of each individual operation both in terms of surgical technique and in terms of the biological and anatomical characteristics of the repaired tissues. Only through the integration of this information with data regarding the patientâ€™s lifestyle and expecta-tions will it be possible to establish a rehabilitation pro-gramme which, though personalised as regards the exec-utive procedures, cannot ignore the times and processesof biological tissue healing in order to achieve the best possible result both in terms of functional recovery and of management of the symptoms | | |
| Pegreffi 2011, Italy | | This study presents a rationale to consider nonoperative treatment an effective option when dealing with patients with rotator cuff tears | Text and opinion | NA | N/A | | N/A | | Rotator cuff tear | | N/A | | N/A | | N/R | | | ..there are patients who, for a variety of reasons, age level of activity, and cuff lesion type, have rotator cuff tears that lend themselves to conservative treatment. | | |
| Cautiero 2012, Italy | | To evaluate the mid-term efficacy of the Lyon hydrotherapy program in the treatment of adhesive capsulitis. | NR | 250 | N/R | | N/R | | Adhesive capsulitis | | NR | | NR | | NR | | | The Lyon hydrotherapy rehabilitation program results in good outcome about pain and range of motion. The mid-term results regarding objective shoulder scores and patient satisfaction are high. The failure rate was low, so this non-operative intervention may be considered for patients affected by adhesive capsulitis. | | |
| Cantarero-Villanueva 2012, Spain | | To evaluate the effectiveness of an 8-week water physical therapy program, focused on low-intensity exercises, on cervical-shoulder pain, pressure pain hypersensitivity, and the presence of active TrPs in a population of breast cancer survivors. | Randomised controlled trial | 66 (water group 33; control 33) | 33f (water group)  33f (control group) | | water grp: 48 yrs (+/-8), Control grp: 47 yrs (+/-9) | | Cervical and shoulder pain, pressure sensitivity, and the presence of triggerpoints (TrPs) in breast cancer survivors | | NR | | NR | | Mean 9 months post-surgery (+/-3) | | | An 8-week water physical therapy program using low-intensity exercise and stretching exercises was effective for improving neck and shoulder/axillary pain and reducing the presence of TrPs in breast cancer survivors as compared with usual care. No significant changes in widespread pressure pain hyperalgesia were found. Our results support that physical therapy interventions maybe clinically useful for avoiding persistent pain and sensory disturbances in breast cancer survivors.  Futures studies are needed to determine the long-term effects of physical therapy in sensory disturbances in patients with breast cancer. | | |
| Subasi 2012, Turkey | | Compare the effectiveness of water- and land-based exercises in patients with shoulder impingement syndrome. | Randomised controlled trial | 57 - land group (28), water group (29 | LG: 7m, 21f; WG: 14m, 15f | | LG 58.3+/-8.6 years,  WG 56.2+/-11.3 years | | Shoulder impingement syndrome (SIS) | | NR | | NR | | LG: 8.9+/-7.5 months WG: 10+/-13.2 months | | | A greater improvement in pain and functional capacity was achieved with combination of physical therapy and water-based exercise program in patients with subacromial impingement syndrome. | | |
| Leonidou 2014, UK | | The aim of this paper is to present the results of MUA and injection of local anaesthetic and corticosteroid for secondary frozen shoulder following breast cancer treatment, and to compare them with the results of MUA in a control group with frozen shoulder | Case control study | 7 | 7f | | Between 41-60 (mean age 48) | | Secondary frozen shoulder after breast cancer treatment | | N/R | | N/R | | 40-100 weeks (mean 54 weeks) | | | The results of this preliminary study suggest that MUA, corticosteroid injection and subsequent physiotherapy have achieved good final results in a series of patients with frozen shoulder secondary to breast cancer treatment. Members of the multidisciplinary team looking after breast cancer patients should be aware of this management option and, on manifestation of this pathology, should refer the patient to an orthopaedic surgeon. | | |
| Killian 2014, USA | | NR | Text and opinion | N/A | N/A | | N/A | | Rotator cuff repair | | N/A | | N/A | | Once incisions have closed - week 3 | | | Early active movement under water is safe (week 3-7 post-op). caution required with increasing speed in water. hydrotherapy can be beneficial in reducing pain, variety of rehab and increased patient confidence from buoyancy of water. | | |
| Bernhardsson 2015, Sweden | | To investigate clinical physiotherapy practice patterns in treating patients with LBP and other common musculoskeletal complaints, and to examine the extent to which reported interventions were supported by research | Cross sectional study | 271 (65% response rate) | 67 m (24.7%), 204 f (75.3%) | | 20-29: 33 (12.2%); 30-39: 74 (27.3%); 40-49: 87 (32.1%): 50-59: 64 (23.6%); >60: 13 (4.8%) | | Subacromial pain | | NR | | NA | | N/R | | | NO CONCLUSIONS RELEVANT TO AQUATIC THERAPY | | |
| Burmaster 2016, USA | | To detail and describe the implementation of a unique comprehensive evidence-based aquatic-assisted rotator cuff repair rehabilitation protocol for a medium size supraspinatus tear | Case report | 1 | 1f | | 73 | | medium size full thickness tear of the supraspinatus tendon, a type III acromion and subacromial bursitis | | N/R | | Cervical fusion Fibromyalgia | | 2 years | | | Safe inclusion of low aquatic exercises as an adjunct to on land treatment programmes for small to medium cuff repairs.  Further studies needed of long term effectiveness. | | |
| Thomson 2016, UK | | The purpose of this systematic review was to provide guidance as to the most effective post RC surgery rehabilitation protocol and investigate factors that may influence outcome. | Systematic review | N/A | N/A | | N/A | | Surgical repair of the rotator cuff | | N/A | | N/A | | N/A | | | Aquatic training in one included study, no conclusions related to hydrotherapy | | |
| Smith 2017, UK | | The purpose of this study was to  (i) develop a tool to (ii) quantify the nature of physiotherapy treatment of people with SIS/RCT in a secondary care, NHS setting | NR | 76 | NR | | NR | | subacromial impingement syndrome/rotator cuff tendinopathy (SIS/RCT). | | NR | | NR | | NR | | | This study provides evidence that this tool has the potential to inform future pragmatic trial design and reporting. Where previous trials have applied specific or narrow treatment approaches then caution must be applied when implementing their findings given the highly varied nature of routine practice. | | |
| Szekeres 2017, Canada | | The objective of this study was to compare the immediate effects of hot packs and whirlpool on hand volume for patients with distal radius fracture (DRF) and to determine whether any changes in volume between these modalities were still present 30 minutes after heat application. Finally, to determine whether there were any differences in volume change between groups after 3 repeated therapy visits. | Randomised controlled trial | 60, therapeutic whirlpool (n=30), moist hotpack (n=30) | WG 4m, 26f; HP 7m, 23 f | | WG 52.7+/-16.1; HP 54.4 +/-11.3 | | Distal radius fracture - non surgical & surgical management | | N/R | | N/R | | WG 40 +/-11.8 days; H{ 39.8 +/-13/2 days | | | Whirlpool is a potential consideration when selecting a heat modality for patients with DRF. | | |
| Enblom 2018, Sweden | | To describe the duration and frequency of participation in water-exercising, and factors experienced to be of significance for breast cancer survivors to continue with water-exercising long-term after breast cancer surgery. | Mixed methods | 29 | 29f - quantitative 24F - qualitative | | median age 66 (42 - 82 yrs range, IQR 61-79.5) | | pain, weakness and stiffness in arm post breast cancer treatment +/- other arm conditions (such as fracture, arthritis, surgery, carpal tunnel, cervical pain, tendinitis) | | NR | | Other arm conditions which affect the arm function:  Wrist or hand fracture -5  Arthritis - 5  Surgery of the shoulder - 1  CTS - 2 Cervical pain disorders - 12 Tendinitis - 2 | | median time since surgery 13 yrs (IQR 3-31.5) | | | Three quarters of the survivors had participated in water-exercising once a week at least half of the median thirteen years since their breast cancer surgery. The factors experienced to be of significance to continue with water-exercising were the convenience of exercising in water, the social interaction within the group, and the access to private dressing rooms | | |
| Oh 2018, Republic of Korea | | The primary purpose of the current study was to assess the effect of 8 weeks of aquomanual therapy on the pain and physical function of patients with chronic musculoskeletal disorders.  The secondary purpose of the study was to investigate factors of the in-depth interview that influence participants to change their lives and verify whether the aquomanual therapy was effective at motivating patients with chronic musculoskeletal disorders. | Mixed-methods | 10 | 4m, 6f | | 26.6 +/- 5.92 years | | Chronic shoulder pain  Neck & shoulder pain (n=1); Shoulder and back pain (n=1); neck, shoulder and back pain (n=5) | | NR | | NR | | Chronic  <3 years to >10 years range - all participants | | | Aquomanual therapy may be helpful for reducing neck, shoulder, and back pain and increasing ROM and QOL in patients with chronic musculoskeletal disorders. Furthermore, participants expressed positive opinions about aquomanual therapy through in-depth interviews, including: 1) decreased pain; 2) increased relaxation and relief of tired-ness; 3) ease of use; 4) understanding and use of the right posture; and 5) novelty and enjoyment | | |
| Levy 2018, UK | | Describes non-operative treatment for massive irreparable or recurrent rotator cuff focusing on deltoid re-education | clinical commentary | NA | NA | | NA | | Massive rotator cuff tear | | NA | | NA | | N/R | | | Rehabilitation for rotator cuff tears is a valid and successful treatment modality. | | |
| Szekeres 2018, Canada | | To investigate the immediate effects of using a moist hot pack (MHP) vs therapeutic whirlpool bath (WB) for improving wrist ROM during a therapy session for patients with distal radius fracture | Randomised controlled trial | 60, 30 MHP, 30 WB | MHP 23f, 7m; WB 26f/4m | | MHP 54.4 +/-11.3; WB 52.7 +/-16.1 | | Distal radius fracture - - non surgical and surgical management | | N/R | | N/R | | 40 days (SD 11.8) | | | Individuals who received WB showed a statistically greater increase in wrist ROM than those receiving MHP during a therapy session, although the difference between groups may or may not be clinically important considering the small changes in ROM observed in this study. Both whirlpool bath and moist heat packs increased ROM so both are suitable for clinical use. | | |
| Roskin 1999, USA | | Focuses on the accelerated rehabilitation of common shoulder surgical procedures | Text and opinion | N/A | N/A | | N/A | | Subacromial decompression  Unilateral and multidirectional instability surgery  Rotator cuff repair. Labral repair | | N/A | | N/A | | 7-10 days postoperatively once sutures are removed and wounds totally healed for subacromial decompression & unidirectional & rotator cuff repair 3 weeks - multidirectional instability  Labral repair - from week 4 | | | The protocols we employ are aggressive but our patients have experienced good success with minimal post-op complications. | | |
| Seitz 2012, USA | | A detailed approach to the phases of rehabilitation, nuances based on the type of arthroplasty, and limitations based on the initial pathology and specifics of arthroplasty are discussed. | Text and opinion | N/A | N/A | | N/A | | Shoulder arthroplasty | | N/A | | N/A | | 6 weeks post surgery | | | When the patient is healthy (regardless of age) with good bone stock, viable musculature, and a good sense of understanding and ability to comply, a near-normal shoulder function can and frequently does result as the outcome from a well-performed arthroplasty and carefully guided rehabilitation program. Conversely, in a frail patient with limited bone stock, poor musculature, and/or deficient rotator cuff tissue with limited rehabilitation potential, pain relief with functional use at or up to the horizontal level and independence in ADL should be the predominant goal. | | |
| MoraFernandez 2021, Spain | | NR | NR | 16 | NR | | N/R | | Breast cancer - mastectomised arm | | NR | | NR | | NR | | | Deep Water Running may significantly improve body composition, muscle strength and range of motion of the operated arm in breast cancer survivors after 12 weeks of regular training. | | |
| Hodgson 2006, UK | | The primary aim of the literature review is to determine the optimum rehabilitation program for conservatively treated proximal humerus fractures based on current research evidence. Fundamental to this question is the role of immobilization in treating these fractures and the secondary aim is to establish if immobilization is necessary for these fractures before rehabilitation starts. The survey aims to establish current clinical practice in the rehabilitation of two part proximal humerus fractures in the UK. The objectives are: (1) Are patients routinely immobilized following a two-part proximal humerus fracture? (2) If, so for how long? (3) When are patients referred for rehabilitation? | literature review & survey | N/A | NA | | NA | | Proximal humerus fracture | | NA | | NA | | NA | | | NR | | |
| Mur-Gimeno 2022< Spain | | In this systematic review, we aimed to synthetize the existing evidence related to the effects of aquatic therapeutic exercise on the following outcomes associated with BC side effects: pain, shoulder mobility, lymphedema, cardiorespiratory fitness, muscle strength, body composition, pulmonary function, cancer-related fatigue and health-related quality of life, both compared to usual care or land-based exercise interventions. As secondary objectives, we aimed to (1) examine adherence to the aquatic therapeutic exercise interventions and (2) explore which exercise parameters (intensity, modality, frequency of training, etc.) may be more effective to improve the aforementioned outcomes in this population. | Systematic review | N/A | N/A | | N/A | | Breast cancer | | N/A | | N/A | | N/A | | | In conclusion, this systematic review found that aquatic therapeutic exercise interventions for BC survivors using a combination of endurance, strength, mobility, stretching and breathing exercises resulted in moderate to large improvements in common side effectsof BC and BC treatments such as pain, decreased range of motion, impaired HRQoL and cardiorespiratory fitness. Unfortunately, largevariations were found in terms of exercise prescription parameters as well as modality of aquatic exercise; thus, specific recommendations for clinical practice are unable to be made at this point. | | |
| Lopez-Zamora 2023, Spain | | N/R | Case report | 1 | 1f | | 57 | | Rotator cuff repair using trapezius & achilles graft | | N/R | | N/R | | 15 days | | | The early inclusion of conventional and aquatic physiotherapy for lower trapezius transfer cases could reduce the functional recovery time of the shoulder. | | |
| Cikes 2023, Switzerland | | The purpose of this study was to compare the clinical and functional outcomes of arthroscopic rotator cuff repair over a 2 years period using 3 post-operative rehabilitation modalities; aquatic therapy, land-based therapy and self-rehabilitation therapy. | Prospective | Aquatic therapy n=54, land-based therapy n=, 57 self rehabilitation n=55 | 30m. 24f | | Aquatic therapy: 56.4 SD 5(46-67) | | Small to medium arthroscopic rotator cuff repair | | N/R | | Smoking status | | 4 weeks post surgery | | | Aquatic therapy has a positive effect on clinical outcomes at 3 months after surgery, but yileds no relevant improvements on function or satisfaction at 1-2 years | | |
| Capdevila-Pons 2023, Spain | | Describes the design of an early, intensive and complementary aquatic rehabilitation protocol after arthroscopic rotator cuff repair based on the Delphi method | Delphi | 12 | 10f 2m | | mean 50, range 34-65 | | Rotator cuff repair | | NR | | NA | | 2nd or 3rd week after surgery (according to the decision of surgeon & healing of incisions) | | | Experts agreed on the early, intensive and complementary aquatic rehabilitation protocol following arthroscopic repair of the rotator cuff. | | |
|  |  | | | | |  | |  | |  | |  | |  | |  |  | |  |  |
|  |  | | | | |  | |  | |  | |  | |  | |  |  | |  |  |
|  |  | | | | |  | |  | |  | |  | |  | |  |  | |  |  |
|  |  | | | | |  | |  | |  | |  | |  | |  |  | |  |  |
|  |  | | | | |  | |  | |  | |  | |  | |  |  | |  |  |
|  |  | | | | |  | |  | |  | |  | |  | |  |  | |  |  |
|  |  | | | | |  | |  | |  | |  | |  | |  |  | |  |  |
|  |  | | | | |  | |  | |  | |  | |  | |  |  | |  |  |
|  |  | | | | |  | |  | |  | |  | |  | |  |  | |  |  |
|  |  | | | | |  | |  | |  | |  | |  | |  |  | |  |  |
