# Supplementary material for: Aquatic exercise interventions in the treatment of musculoskeletal upper extremity disorders: A scoping review
Source: Clin Rehabil. 2025 Feb 2;39(5):565–79. doi: 10.1177/02692155251315078 (PMC12099020; doi:10.1177/02692155251315078)
Supplement: sj-docx-7-cre-10.1177_02692155251315078 - Supplemental material for Aquatic exercise interventions in the treatment of musculoskeletal upper extremity disorders: A scoping review [file sj-docx-7-cre-10.1177_02692155251315078.docx]

|  | **Brief name** | **Why** | **What** | | **Who** | **How** | **Where** | **When & how much** | **Tailoring** | **Modifications** | **How well** | |
| --- | --- | --- | --- | --- | --- | --- | --- | --- | --- | --- | --- | --- |
| **Study ID** |  |  | **Materials** | **Procedures** |  |  |  |  |  |  | **Planned** | **Actual** |
| Delbrouck 2003 | FR | FR | NR | NR | NR | NR | PR | PR | NR | NR | NR | NR |
| Castelhano 2022 | FR | NR | NR | NR | NR | NR | NR | NR | NR | NR | NR | NR |
| Klintberg 2009 | FR | FR | NR | PR | FR | PR | PR | PR | NR | NR | NR | NR |
| Zanazzo 2014 | FR | NR | NR | NR | NR | NR | NR | PR | NR | NR | NR | NR |
| Grigware 2015 | FR | FR | FR | PR | NR | FR | NR | PR | NR | NR | NR | PR |
| Appunni 2012 | FR | FR | NR | PR | FR | FR | FR | NR | NR | NR | NR | PR |
| Huss 2014 | FR | NR | NR | NR | NR | PR | NR | PR | NR | NR | NR | NR |
| Yeomans 2012 | FR | FR | NR | NR | NR | NR | PR | PR | NR | NR | NR | NR |
| Turnbull 2016 | FR | FR | FR | PR | FR | PR | PR | PR | NR | NR | NR | NR |
| Maynard 2000 | FR | NR | FR | FR | NR | PR | NR | PR | NR | FR | NR | NR |
| Fisken 2015 | FR | FR | FR | FR | FR | FR | PR | PR | NR | NR | NR | NR |
| Clark 2011 | FR | FR | FR | PR | FR | PR | NR | PR | NR | NR | NR | NR |
| Palmer 1998 | FR | FR | FR | FR | FR | PR | PR | PR | FR | NR | NR | NR |
| Gutierrez-Espinoza 2019 | FR | FR | FR | PR | NR | PR | FR | FR | NR | NR | NR | NR |
| Gliga 2022 | FR | FR | FR | FR | NR | NR | FR | PR | PR | NR | NR | NR |
| Dufournet 2022 | FR | FR | NR | PR | FR | PR | PR | NR | NR | NR | NR | NR |
| Hoyrup 1986 | FR | FR | FR | FR | NR | FR | NR | PR | NR | NR | NR | NR |
| Revay 1992 | FR | NR | FR | FR | NR | FR | PR | PR | NR | NR | NR | NR |
| Sokk 2007 | FR | NR | NR | NR | FR | PR | PR | PR | FR | NR | NR | NR |
| Brady 2008 | FR | FR | FR | PR | FR | FR | NR | PR | NR | NR | NR | PR |
| Janssen 2009 | FR | FR | FR | FR | NR | NR | NR | FR | NR | NR | NR | NR |
| Cautiero 2012 | FR | NR | NR | NR | NR | NR | NR | PR | NR | NR | NR | NR |
| Cantarero-Villanueva 2012 | FR | FR | FR | PR | FR | FR | PR | FR | FR | NR | FR | FR |
| Subasi 2012 | FR | FR | FR | PR | FR | PR | PR | NR | NR | NR | NR | NR |
| Leonidou 2014 | FR | NR | NR | NR | NR | PR | NR | PR | NR | NR | NR | NR |
| Burmaster 2016 | FR | FR | FR | FR | FR | NR | PR | PR | PR | NR | FR | NR |
| Enblom 2018 | FR | FR | NR | FR | FR | FR | PR | PR | NR | NR | NR | FR |
| Oh 2018 | FR | FR | FR | FR | FR | FR | PR | PR | NR | NR | NR | NR |
| Szekeres 2018 |  |  |  |  |  |  |  |  |  |  |  |  |
| Szekeres 2017 | FR | FR | FR | FR | FR | FR | FR | PR | NR | NR | NR | NR |
| MoraFernandez 2021 | FR | FR | NR | FR | NR | FR | NR | PR | NR | NR | NR | NR |
| Lopez-Zamora 2023 | FR | FR | NR | PR | NR | NR | NR | PR | NR | NR | NR | NR |
| Cikes 2023 | FR | FR | FR | PR | FR | FR | PR | PR | NR | NR | NR | PR |
| Smith 2017 | FR | NR | NA | NA | NA | NA | NA | NA | NA | NA | NA | NA |
| Bernhardsson 2015 | FR | NR | NA | NA | NA | NA | NA | NA | NA | NA | NA | NA |
|  |  |  |  |  |  |  |  |  |  |  |  |  |
| **Totals** |  |  |  |  |  |  |  |  |  |  |  |  |
| FR | 34 (100%) | 24 (75%) | 18 (56%) | 12 (37.5%) | 16 (50%) | 12 (37.5%) | 4 (12.5%) | 3 (9.4%) | 3 (9.4%) | 1 (3.1%) | 2 (6.2%) | 2 (6.2%) |
| PR | 0 | 0 | 0 | 12 (37.5%) | 0 | 11 (34.3%) | 15 (46.9%) | 25 (78.1%) | 2 (6.2%) | 0 | 0 | 4 (12.5%) |
| NR | 0 | 10 (25%) | 14 (44%) | 8 (25%) | 16 (50%) | 9 (28.2%) | 13 (40.6%) | 4 (12.5%) | 27 (84.4%) | 31 (96.9%) | 30 (93.8%) | 26 (81.3%) |
| NA | 0 | 0 | 2 | 2 | 2 | 2 | 2 | 2 | 2 | 2 | 2 | 2 |

Key: FR – fully reported; PR – Partially reported; NR – Not Reported; NA – Not Applicable
