# Supplementary material for: Aquatic exercise interventions in the treatment of musculoskeletal upper extremity disorders: A scoping review
Source: Clin Rehabil. 2025 Feb 2;39(5):565–79. doi: 10.1177/02692155251315078 (PMC12099020; doi:10.1177/02692155251315078)
Supplement: sj-docx-8-cre-10.1177_02692155251315078 - Supplemental material for Aquatic exercise interventions in the treatment of musculoskeletal upper extremity disorders: A scoping review [file sj-docx-8-cre-10.1177_02692155251315078.docx]

Table 2: Aquatic therapy exercise types

| Type of Exercise | Shoulder | Elbow | Forearm/Wrist/Hand |
| --- | --- | --- | --- |
| ROM |  |  |  |
| Passive | 3 |  |  |
| Active assisted | 10 |  |  |
| Active | 4 | 1 | 5 |
| ROM (type not specified) | 6 |  |  |
| Stretches | 7 |  |  |
| Resistance / strengthening | 14 | 2 | 1 |
| Proprioceptive | 3 |  |  |
| PNF/Bad Regaz | 2 |  |  |
| Functional | 2 |  |  |
| Deep water running / aerobic | 3 |  |  |
