# Supplementary material for: Aquatic exercise interventions in the treatment of musculoskeletal upper extremity disorders: A scoping review
Source: Clin Rehabil. 2025 Feb 2;39(5):565–79. doi: 10.1177/02692155251315078 (PMC12099020; doi:10.1177/02692155251315078)
Supplement: sj-docx-9-cre-10.1177_02692155251315078 - Supplemental material for Aquatic exercise interventions in the treatment of musculoskeletal upper extremity disorders: A scoping review [file sj-docx-9-cre-10.1177_02692155251315078.docx]

Table 3: Upper extremity domains and outcome measures

| **Domain** | **Frequency of domain usage** | **Outcome measures (OM) associated with domain** | **Frequency of OM use** | **Number of OM per domain** | **Body area** |
| --- | --- | --- | --- | --- | --- |
| **ROM** | **21 ROM domains in 21 studies** |  |  | **3** |  |
| Shoulder PROM | 2 | Inclinometer | 1 |  | Shoulder |
|  |  | N/R | 1 |  | Shoulder |
| Shoulder AROM | 6 | Goniometer | 3 |  | Shoulder |
|  |  | N/R | 3 |  | Shoulder |
| Shoulder AROM & PROM | 4 | Goniometer | 3 |  | Shoulder |
|  |  | N/R | 1 |  | Shoulder |
| Shoulder ROM (AROM/PROM not specified) | 5 | Goniometer | 1 |  | Shoulder |
|  |  | N/R | 4 |  | Shoulder |
| Shoulder AROM, PROM & functional ROM | 1 | Hand behind back (HBB), Hand behind head (HBH) | 1 |  | Shoulder |
|  |  | Goniometer | 1 |  | Shoulder |
| Elbow ROM (AROM/PROM not specified) | 1 | N/R | 1 |  | Arm / multiple |
| Wrist AROM | 1 | Goniometer | 1 |  | Wrist |
| Total active movement | 1 | Goniometer | 1 |  | Hand |
| **Pain** | **20 pain domains in 19 studies** |  |  | 6 |  |
| Pain | 19 | NPRS | 2 |  | Shoulder |
|  |  | VAS (including one measure of pain at rest & activity) | 14 |  | Shoulder (n=10), wrist & hand (n=1), wrist (n=1), hand (n=1) |
|  |  | N/R | 1 |  | Shoulder |
|  |  | Arthritis Impact Measurement Scale 2-Short form (AIMS-SF) | 1 |  | Hand |
|  |  | Shoulder Pain & Disability Index (SPADI) | 2 |  | Shoulder |
|  |  | Pain Disability Questionnaire (PDQ) | 1 |  | Shoulder |
| **Pressure pain sensitivity** | 1 | Pressure pain threshold | 1 |  | Shoulder |
| **Function** | **9** | Hand in neck, hand in back and pour out of a pot | 1 | **17** | Shoulder |
|  |  | Constant-Murley Score | 2 |  | Shoulder |
|  |  | Isometric shoulder strength (Isobex dynamometer) | 1 |  | Shoulder |
|  |  | Functional Index of the Shoulder | 1 |  | Shoulder |
|  |  | Difficulty with personal care, household tasks, driving, recreation and socialisation expressed by patient | 1 |  | Shoulder |
|  |  | Disabilities of the Arm, Shoulder and Hand (DASH) (including QuickDASH (shortform) n=1) | 3 |  | Shoulder (n=2), wrist & hand (n=1) |
|  |  | Patient-Rated Wrist Evaluation (PRWE) questionnaire | 1 |  | Wrist & hand |
|  |  | American Shoulder and Elbow Surgeons (ASES) score | 1 |  | Shoulder |
|  |  | Simple Shoulder Test (SST) | 1 |  | Shoulder |
|  |  | Single Assessment Numeric Evaluation (SANE) | 1 |  | Shoulder |
|  |  | Subjective assessment of 9 activities of daily living on a 5-point scale & 4 upper limb movements measured on a 6-point scale | 1 |  | Shoulder |
|  |  | Shoulder Pain & Disability Index (SPADI) | 2 |  | Shoulder |
|  |  | Western Ontario Rotator Cuff (WORC) Index | 1 |  | Shoulder |
|  |  | Functional limitations | 1 |  | Shoulder |
|  |  | Subjective Shoulder Value (SVV) | 1 |  | Shoulder |
|  |  | Pain Disability Questionnaire (PDQ) | 1 |  | Shoulder |
|  |  | Penn Shoulder Score (PSS) | 1 |  | Shoulder |
| **Strength** | **9 strength domains in 7 studies** |  |  | **3** |  |
| **Isokinetic shoulder strength** | 1 | KIN-Com dynamometer | 1 |  | Shoulder |
| **Isometric shoulder strength** | 4 | Dynamometer (microfet (n=1), isobex (n=1), Chatillon® strain gauge (n=1), handheld dynamometer (n=1)) | 4 |  | Shoulder |
| **Hand grip strength** | 4 | Dynamometer (JAMAR (n=2), handheld dynamometer (n=1) | 3 |  | Shoulder (n=1), wrist & hand (n=1), hand (n=1) |
|  |  | N/R | 1 |  | Shoulder |
| **Adverse effects / events /complications** | **7** |  |  | **5** |  |
|  |  | Number of patients with further/subsequent shoulder problems | 3 |  | Shoulder |
|  |  | Number of patients requiring further orthopaedic intervention | 1 |  | Shoulder |
|  |  | Number of adverse events | 3 |  | Shoulder |
|  |  | Number of post operative complications | 1 |  | Shoulder |
|  |  | Number of repeat MUA's | 1 |  | Shoulder |

| HRQoL | 5 | Western Ontario Rotator Cuff (WORC) Index | 2 | 5 | Shoulder |
| --- | --- | --- | --- | --- | --- |
|  |  | Arthritis Impact Measurement Scale 2-Short form (AIMS-SF) | 1 |  | Hand |
|  |  | EQ barometer | 1 |  | Shoulder |
|  |  | EQ-5D | 1 |  | Shoulder |
|  |  | Short Form 36 Health Status Questionnaire (SF-36) | 1 |  | Shoulder |

| **Satisfaction** | **4** | Likert scale - 5 options | 1 | **3** | Shoulder |
| --- | --- | --- | --- | --- | --- |
|  |  | Very satisfied, satisfied, unsatisfied | 1 |  | Shoulder |
|  |  | 0-3 scale | 1 |  | Shoulder |
|  |  | N/R | 1 |  | Shoulder |
| **Hand volume** | **3** | Volume of water displacement measured using a hand volumeter & graduated cylinder | 3 | **1** | Wrist & hand (n=1), Wrist (n=1), hand (n=1) |
| **Disability** | **1** | Shoulder Pain & Disability Index (SPADI) | 1 | **1** | Shoulder |
| **Joint positional sense** | **1** | Kinesimeter | 1 | **1** | Shoulder |
| **Tendon healing** | **1** | Ultrasound Sugaya classification | 1 | **1** | Shoulder |
| **Endurance (isometric)** | **1** | Handheld dynamometer - 30% MVC until exhaustion | 1 | **2** | Shoulder |
|  |  | EMG activity | 1 |  | Shoulder |
| **Trigger point exploration** | **1** | Presence of active muscle trigger points | 1 | **1** | Shoulder |
| **Self-efficacy** | **1** | Perceived Wellness Survey (PWS) | 1 | **1** | Shoulder |
| **Duration & frequency** | **1** | Number of semesters | 1 | **1** | Shoulder |
| **Fatigue** | **1** | Fatigue severity scale | 1 | **1** | Shoulder |
| **Domain N/R** | **13** | Oxford Shoulder Score (OSS) | 2 | **10** | Shoulder |
|  |  | Simple Shoulder Test (SST) | 2 |  | Shoulder |
|  |  | Disabilities of the Arm, Shoulder and Hand (DASH) | 3 |  | Shoulder (n=1). Arm / multiple (n=1), wrist (n=1) |
|  |  | Constant-Murley Score (Constant score (n=2), Modified Constant score(n=1)) | 3 |  | Shoulder |
|  |  | Task related patient goals | 1 |  | Shoulder |
|  |  | Workstop duration | 1 |  | Shoulder |
|  |  | Minnesota Leisure Time Physical Activity Questionnaire | 1 |  | Shoulder |
|  |  | Patient-Rated Wrist Evaluation (PRWE) questionnaire | 1 |  | Wrist |
|  |  | Subjective Shoulder Value (SVV) | 1 |  | Shoulder |
|  |  | Measure yourself Medical Outcome Profile (MYMOP) | 1 |  | Shoulder |
|  |  | N/R | 1 |  | Shoulder |
